# Body composition of children born HIV-exposed uninfected in rural Zimbabwe

**DOI:** 10.1371/journal.pone.0319542

**Published:** 2026-09-11

**Authors:** Joe D. Piper, Robert Ntozini, Idah Mapurisa, Tsitsi Mashedze, Clever Mazhanga, Florence D. Majo, Bernard Chasekwa, Naume V. Tavengwa, Kim Michaelsen, Jean Humphrey, Jonathan Wells, Andrew J. Prendergast

**Affiliations:** 1 Zvitambo Institute for Maternal and Child Health Research, Harare, Zimbabwe; 2 Blizard Institute, Queen Mary University of London, London, United Kingdom; 3 Department of Nutrition, Exercise and Sports, University of Copenhagen, Copenhagen, Denmark; 4 Johns Hopkins Bloomberg School of Public Health, Baltimore, Maryland, United States of America; 5 Population Policy and Practice Research and Teaching Department, UCL Great Ormond Street Institute of Child Health, London, United Kingdom; University of Cape Town, SOUTH AFRICA

## Abstract

**Background:**

Globally, 15 million children were born HIV-exposed uninfected (CHEU) from mothers living with HIV. CHEU have increased mortality, morbidity and stunting compared to children HIV-unexposed (CHU). Our objective was to compare body composition between CHEU and CHU, which may underlie this vulnerability.

**Methods:**

In a substudy among 223 children (36 CHEU and 187 CHU) enrolled in the Sanitation Hygiene Infant Nutrition Efficacy (SHINE) trial in rural Zimbabwe, we measured height, knee-heel length, weight, calf circumference, skinfold thicknesses (triceps, subscapular and calf), and bio-electrical impedance at 2 years of age. Generalised estimating equations were used to compare body composition between CHEU and CHU, and interactions with linear growth and birthweight.

**Results:**

CHEU had significantly lower length-for-age, knee-heel length, weight-for-age, mid-upper arm circumference and calf circumference compared to CHU. CHEU had a lower impedance index (adjusted mean difference −0.49, 95% CI −0.87, −0.11), reflecting less lean mass, and lower skinfold thicknesses (subscapular Z-score adjusted mean difference −0.49 (95% CI −0.97, −0.02); triceps Z-score −0.48 (95% CI −0.83, −0.13)), indicating reduced subcutaneous fat. In early-life, CHEU had lower birthweight. However, no significant interaction between stunting or birthweight and HIV exposure was observed for body composition outcomes.

**Conclusion:**

CHEU have a distinct pattern of body composition by 2 years of age, characterised by reduced lean and fat mass compared to CHU which was not explained by reduced linear growth or lower birthweight. This pattern of early-life growth may contribute to the increased vulnerability of CHEU to mortality, morbidity and reduced neurodevelopment.

## Introduction

The success of prevention of mother-to-child transmission (PMTCT) of HIV has driven an increase in the number of children who are HIV-exposed uninfected (CHEU) [1]. By 2021, the number of CHEU was estimated at 16 million globally, and this population continues to grow [2]. In sub-Saharan Africa, CHEU are more likely to be born premature or small-for-gestational age [3], and have poorer postnatal growth compared to children unexposed to HIV [4–6]. In rural Zimbabwe, in the current ART era, half of all CHEU are stunted by 18 months of age [1], compared to ~30% of children who are HIV-unexposed (CHU) [7]. Stunting has effects across the life-course, and is associated with reduced educational attainment [8]. CHEU therefore represent a vulnerable group, who fail to reach their growth and developmental potential in early life.

The causes and consequences of growth failure in CHEU remain poorly understood. Mechanistic insights into the pattern and timing of growth failure would be valuable to inform interventions and to predict long-term health outcomes. Measuring body composition (i.e., fat and lean mass) provides useful insights into the *quality* of growth, and can inform future homeostatic reserve [9]. There is evidence that both fat and lean mass are reduced in stunting [10,11]. Fat mass provides short-term benefits for immune function [11] and survival [12]; however, it also has longer-term metabolic health costs [9]. The distribution of fat within the body is also important: genes associated with immune function are highly expressed in intra-abdominal fat [13] which helps drive inflammation [14]. An ecogeographical analysis reported that central compared to peripheral fat mass is linked more closely with pathogen load across populations, indicating its role in providing energy for immune function [15]. Lean mass is associated with increased organ size [16] and reduced cardio-metabolic risk [17]. Lean mass is also much more closely associated with neurodevelopment than is fat mass [18].

Studies that have performed more detailed body composition among CHEU in low- and middle-income countries (LMIC) have generally shown poorer growth compared to CHU. However, there are differences between studies: the sum of skinfolds was preserved in one study of 295 infants in Kenya [19], but lower skinfold thicknesses among CHEU were found in a study of 238 infants in Uganda [20], where worse food insecurity suggested a prioritisation of central skinfold thickness. In the USA, one cross-sectional study showed lower birthweight and a trend towards decreasing triceps and subscapular skinfold thicknesses in CHEU at 2 years of age, compared to CHU [21]. A recent cross-sectional study of premature CHEU infants from South Africa suggested they were smaller in weight and length at birth, gained weight more slowly and had reduced skinfold thicknesses up to 28 days of age [22]. Follow-up of CHEU birth cohorts at school-age in Zambia suggested lower height, mid-upper-arm and thigh circumference and reduced fat percentages, when estimated with bioimpedance [23]. There is a pressing need for further characterisation of CHEU compared to CHU in sub-Saharan Africa, where the majority of CHEU live, especially during the first two years after birth when stunting prevalence peaks.

Here we report body composition measurements in rural Zimbabwean CHEU compared to CHU at 2 years of age, (the end of the first 1000 days, and a time of peak stunting). These children were recruited to a sub-study within the SHINE cluster-randomised trial in Zimbabwe [24]. We used bio-impedance, skinfold thicknesses, anthropometry and knee-heel length to provide a comprehensive assessment of growth quality and future metabolic disease risk. We hypothesised that CHEU compared to CHU would have worse growth in measures of lean mass (bioimpedance), fat mass (skinfold thicknesses) and relatively shorter legs (knee-heel length), and that these differences would not entirely be explained by lower birthweight and more stunting among CHEU.

## Materials and methods

### Participants

Body composition measurements were performed in a substudy of the Sanitation Hygiene Infant Nutrition Efficacy (SHINE) trial (registered at Clinical trials.gov, NCT01824940). Mothers gave written informed consent to participate in the SHINE trial, and additional consent for the body composition sub-study, which was approved by the Medical Research Council of Zimbabwe (MRCZ/A/1675) and Johns Hopkins Bloomberg School of Public Health (IRB 4205). The design, methods and outcomes of SHINE have been previously reported [7,25,26] and the protocol is available on Open Science Framework (https://osf.io/w93hy/). In brief, SHINE tested the independent and combined effects of a water, sanitation and hygiene (WASH) intervention and an infant and young child feeding (IYCF) intervention on stunting and anaemia at 18 months of age. The study area comprised two rural districts of central Zimbabwe, which were divided into 212 clusters, defined as the catchment area of 1–4 Community Health Workers (CHWs) employed by the Ministry of Health and Child Care (MoHCC). Between November 2012 and March 2015, CHWs identified pregnancies through prospective surveillance; women were eligible to enroll if they permanently resided in one of the rural study clusters, were confirmed pregnant and provided written informed consent. HIV testing, definitions of CHEU and CHU are detailed in supporting information in S1 File.

### Body composition substudy

Children were eligible for the body composition substudy if they i) completed the trial endpoint visit at 18 months of age; ii) lived in the catchment area of the substudy, which comprised approximately one-quarter of the total SHINE area, constituting 52 trial clusters; and iii) were between 102–112 weeks of age during the period of enrolment.

Between 20^th^ September 2016–2^nd^ May 2017, children had a home visit by two trained research nurses to measure anthropometry (height, weight, head circumference and mid-upper arm circumference) using standard methods [7]. Body composition was measured by bio-impedance analysis, skinfold thicknesses using Holtain calipers, and knee-heel length using a knemometer. A locally-built case ensured the equipment was protected whilst being transported between households by motorbike.

### Skinfold thickness

**Fig 1:**
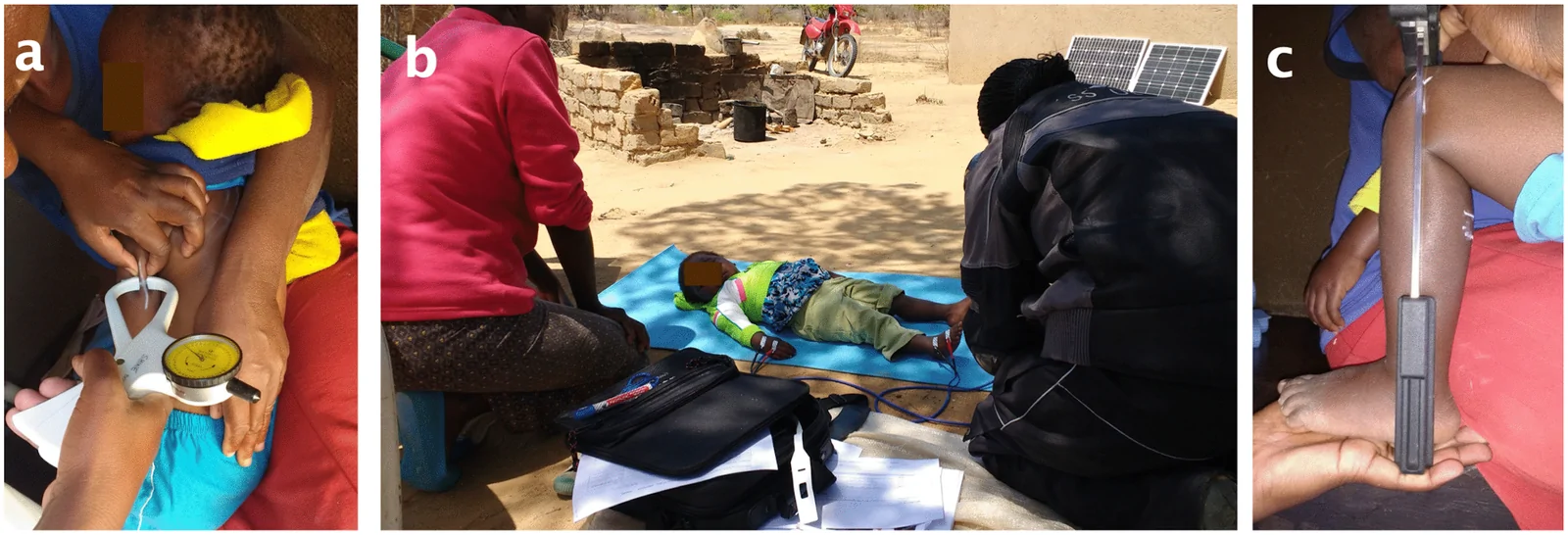
Body composition measurement techniques in rural Zimbabwe. a) Skinfold thickness (here demonstrating triceps skinfold), b) Bioimpedance analysis (BIA) showing electrodes attached to right hand and foot of the child, c) Positioning for knee-heel length measurement. Printed under a CC BY license with permission from JDP who is the original copyright holder of these images.

Skinfold thickness (Fig 1a) measures the subcutaneous fat layer around the body and describes its distribution. Subscapular, triceps and maximal calf skinfold thicknesses were measured to the nearest 0.2 mm using a skinfold caliper (Holtain, Crosswell, Wales) according to a standard operating procedure (SOP), with the median of three readings used. The absolute values of the triceps and calf skinfolds were combined for a composite measure of peripheral fat, whilst subscapular skinfolds provided a central fat measurement. All three skinfolds were summed to provide the total skinfold thickness. In addition, triceps and subscapular Z-scores were calculated based on WHO Reference growth charts (calf skinfolds do not have available growth charts).

### Bio-impedance analysis

Bio-impedance analysis (BIA; Fig 1b) can be used to assess tissue health and estimate lean mass, using an imperceptible electrical signal between the hand and foot [27]. Tissue and cellular health are indicated by the phase angle, which provides an estimate of membrane integrity [28,29]. BIA was measured using the Bodystat 1500 MDD instrument (BodyStat, Douglas, Isle of Man). Once set up at the household, calibration was performed before each set of BIA measurements using the 500 Ohm calibration resistor provided (the acceptable range was 497–503 Ohms). The child was measured lying on a padded mat with arms and legs straight, as previously described [30,31]. The right hand and foot were cleaned and electrodes were attached. There were no restrictions on fluid intake for children to ensure they remained calm. BIA measurements were performed in duplicate according to an SOP.

Raw BIA data were used to generate two rankings of lean mass, and one of cellular quality, as follows. First, an estimate of absolute lean mass was obtained from the impedance index, calculated as (Ht^2^/Z), where Ht is height and Z is impedance measured at 50 kHz. In any population, there is a strong association of Ht^2^/Z with lean mass. If this association is calibrated, using a direct measurement of lean mass as the reference method, lean mass can be predicted from Ht^2^/Z, and fat mass can be estimated as the difference of lean mass and weight. However, in the absence of a calibration equation, population rankings of Ht^2^/Z and lean mass are near-identical, hence the raw data will provide similar epidemiological results [29]. Therefore, in this population, Ht^2^/Z was used as a proxy for lean mass. Second, to correct for body size, lean mass is often adjusted for the square of height to give the lean mass index, expressed in the same kg/m^2^ units as BMI. However, when calculating lean mass index from BIA data, the data are effectively processed as [Ht^2^/Z/Ht^2^], which simplifies to 1/Z. As demonstrated previously, rankings of 1/Z are near-identical to rankings of lean mass index [29], hence 1/Z was used as a proxy for lean mass independent of height. Third, phase angle was also used as a measure of cellular health [29]. Data were excluded if the phase angle (PA) was more than 8^0^, which is considered implausible [32], or if there was poor repeatability (duplicate value differences  > 0.3^0^ for PA, and ≥6.0 Ω for either resistance (R) or reactance (Xc)).

### Knee-heel length

Knee-heel length (Fig 1c) was recorded using a knemometer, with the median of three readings used. Two different types of knemometer were used and their correlation compared on a Bland-Altman plot (S3 Fig in S1 File). Further detail is available in (Supporting information, supplementary methods 1 in S1 File).

### Statistical analysis and sample size

Sample size was determined by the feasibility of recruitment. A detectable effect size of 0.5 SD for differences between CHEU and CHU was estimated with 80% power, assuming 52 clusters and intra-cluster correlation coefficient of 0.05, with alpha 0.05. Analysis was performed using Stata 15.0. Associations between body composition measures and length-for-age Z-score (LAZ) at 24 months were undertaken to explore how body composition varied with linear growth. Then four different models were used to evaluate differences in body composition between CHEU and CHU. Model 1 was an unadjusted analysis using generalised estimating equations (GEE) with an exchangeable working correlation structure to account for clustering. Model 2 was adjusted for trial arm only. Model 3 was adjusted for trial factors affecting body composition measurements (study nurse performing the measurement, exact child age at assessment, and calendar month of birth) in addition to trial arm. Calendar month of birth was used to adjust for seasonality. Model 4 was additionally adjusted for potential socioeconomic and demographic confounders which are not likely to be on the causal pathway leading from HIV exposure to body composition outcomes (see supporting information, Supplementary Material 1 in S1 File).

We also tested whether the associations between HIV exposure and body composition outcomes varied by i) stunting at 2 years, ii) length-for-age Z-score (LAZ) at 2 years and iii) birthweight, using P < 0.1 as evidence of an interaction. Initially, we assumed that body composition increased similarly with linear growth and birthweight across all children. We then examined the interaction between HIV exposure and linear growth (stunting at 2 years) to determine whether the relationship between body composition and postnatal growth differed by HIV exposure status. We conducted a similar analysis for LAZ at 2 years. Finally, we tested for an interaction with birthweight to assess whether the association between body composition and birthweight differed by HIV exposure. These analyses were performed to determine whether differences in body composition among HIV-exposed children could be partly explained by birthweight or postnatal growth.

## Results

Between November 2012 and March 2015, 5280 pregnant women were enrolled from 211 clusters at median 12 (IQR 9, 16) gestational weeks. Of 4845 live births, 4427 children were evaluated at 18 months, including 1004 from the substudy catchment area. At mean age 24 (SD = 0.4) months, 230 children from 52 clusters were enrolled into the body composition substudy between November 2016 and June 2017 [33]. Clusters were defined by the catchment area of 1–4 village health workers, based on the SHINE trial design [34]. Of the 230 enrolled children, 4 were excluded due to unknown maternal HIV status during pregnancy. Of the 226 remaining children, 1 was HIV-positive and 2 had unknown HIV status at 18 months, and were excluded from the analysis, leaving 223 children, of whom 36 were CHEU and 187 CHU (See supporting information, S1 Fig CONSORT diagram). There were no recorded harms from this study.

### Baseline characteristics

Baseline characteristics of children who were enrolled or not enrolled in the body composition sub-study are shown (See supporting information, S1 Table in S1 File). There were no significant differences in distribution by intervention arm, maternal schooling, maternal height, household wealth quintile, infant sex or birthweight. The body composition sub-study enrolled slightly older mothers (27.9 vs 26.1 years), with some differences in the distribution of religions, and a lower Edinburgh Postnatal Depression Scale (0.9 vs 3.2). HIV-positive mothers in the body composition sub-study, compared to HIV-positive mothers not enrolled, had a lower CD4 count during pregnancy (mean 427 vs. 487 cells/mm^3^) but no significant differences in ART regimen. Enrolled households had a lower Coping Strategies Index (mean score 0 compared to 1) than those not enrolled, indicating improved food security. Enrolled children had a marginally higher mean length-for-age Z-score than those not enrolled (−0.78 vs −0.88), but no significant difference in birthweight.

Baseline characteristics of CHEU and CHU enrolled in the body composition substudy, together with their mothers, are shown in Table 1. There was no significant difference in trial arm distribution, household characteristics or maternal characteristics, apart from older maternal age among CHEU versus CHU. Of note, CHEU versus CHU had a significantly lower mean birthweight (2.8 vs. 3.1 kg) with a significantly higher proportion of low birthweight (<2.5 kg) than CHU.

**Table 1:** Baseline characteristics of children born HIV-exposed uninfected (CHEU) compared to children HIV-unexposed (CHU).

	CHEU	CHU	P value
Mothers, N	36	185	
Children, N	36	187	
Trial Arm			
Standard of care	7/36 (19.4%)	59/185 (31.9%)	0.066
IYCF	6/36 (16.7%)	41/185 (22.2%)	
WASH	6/36 (16.7%)	35/185 (18.9%)	
IYCF & WASH	17/36 (47.2%)	50/185 (27.0%)	
Maternal characteristics			
Mean years of schooling completed (SD)	9.9 (1.8)	9.5 (2.0)	0.14
Mean age (SD), years	29.5 (5.5)	27.6 (6.8)	0.023
Median parity (IQR)	2.0 (1.0, 3.0)	2.0 (1.0, 3.0)	0.579
Mean height (SD), cm	158.5 (6.5)	160.0 (9.8)	0.354
Mean mid-upper-arm circumference (SD), cm	26.7 (3.1)	26.7 (3.1)	0.889
Married	33/34 (97.1%)	168/175 (96.0%)	0.78
Employed	4/35 (11.4%)	14/180 (7.8%)	0.498
Religion:			
Apostolic	13/34 (38.2%)	82/177 (46.3%)	0.602
Other Christian	19/34 (55.9%)	88/177 (49.7%)	
Other	2/34 (5.9%)	7/177 (4.0%)	
Mean Edinburgh postnatal depression scale (SD)	0.6 (1.2)	0.9 (2.2)	0.353
Mean CD4 count in pregnancy (SD), cells/µL	426 (202)	N/A	N/A
Documented ART use during pregnancy	33/36 (91.7%)	N/A	N/A
Tenofovir disoproxil fumarate-based ART regimen	24/33 (72.7%)	N/A	N/A
Zidovudine-based ART regimen	4/33(12.1%)	N/A	N/A
Other/unknown ART regimen	5/33 (15.2%)	N/A	N/A
Documented co-trimoxazole prophylaxis during pregnancy	19/36 (52.8%)	N/A	N/A
Household Characteristics			
Median number of occupants [IQR]	4.0 (3.0, 6.0)	4.0 (3.0, 5.0)	0.73
Median Coping Strategies Index score [IQR]	0.0 (0.0, 3.0)	0.0 (0.0, 3.0)	0.869
Wealth Quintile:			
Lowest	10/36 (27.8%)	25/180 (13.9%)	0.313
Second	6/36 (16.7%)	39/180 (21.7%)	
Middle	8/36 (22.2%)	36/180 (20.0%)	
Fourth	6/36 (16.7%)	46/180 (25.6%)	
Highest	6/36 (16.7%)	34/180 (18.9%)	
Electricity in home	0/36 (0.0%)	6/179 (3.4%)	
Improved latrine at household	12/35 (34.3%)	60/179 (33.5%)	0.913
Main source of household drinking water improved	27/35 (77.1%)	122/178 (68.5%)	0.222
Infant characteristics			
Female sex	17/36 (47.2%)	98/187 (52.4%)	0.55
Mean birth weight (SD), kg	2.8 (0.5)	3.1 (0.4)	<0.001
Birth weight <2.5 kg	7/36 (19.4%)	9/184 (4.9%)	0.007
Institutional delivery	31/35 (88.6%)	168/181 (92.8%)	0.39
Vaginal delivery	29/36 (80.6%)	170/184 (92.4%)	0.106

IYCF: Infant and young child feeding, WASH: Water, Sanitation and Hygiene, ART: Anti-retroviral therapy.

### Body composition results

**Fig 2:**
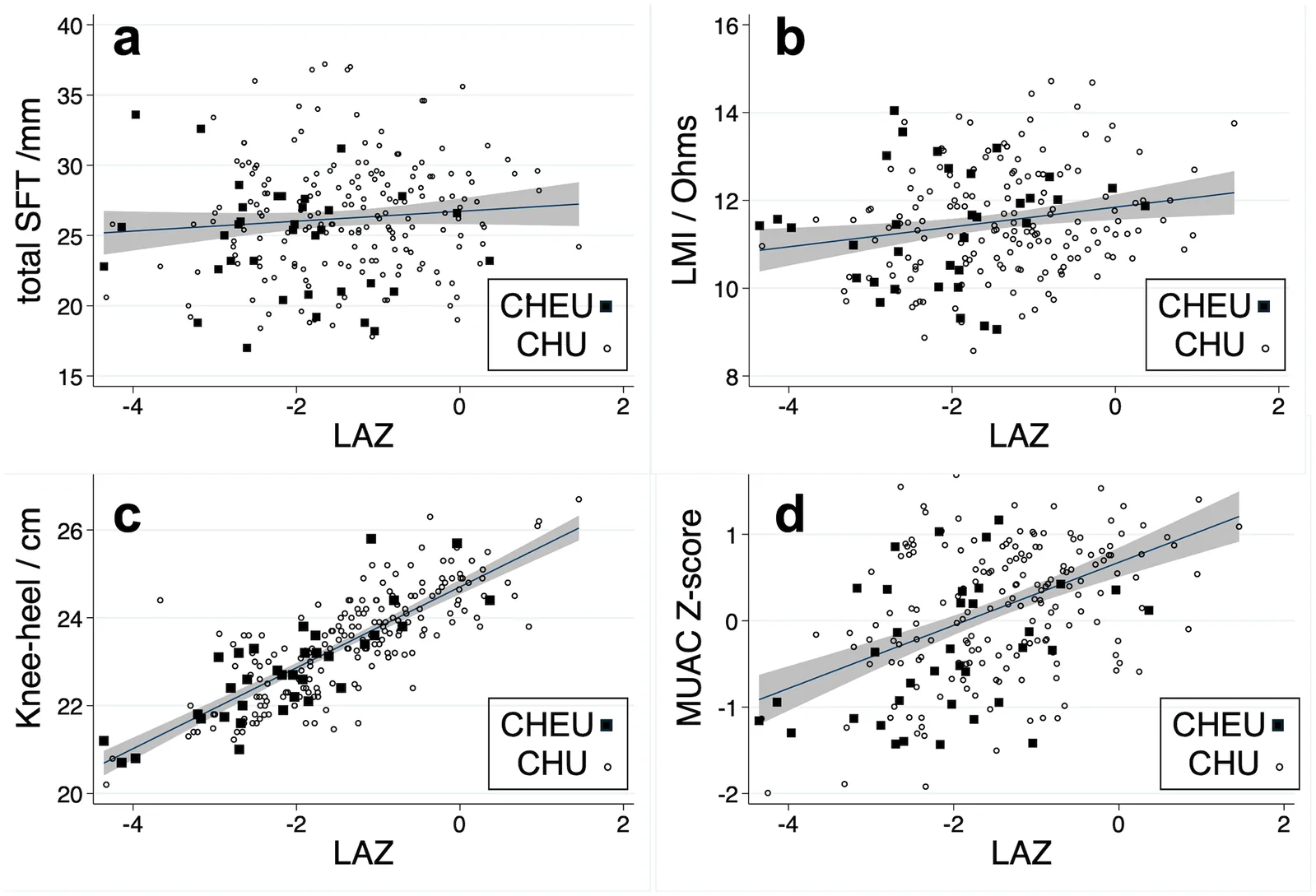
Associations between body composition and LAZ at 2 years of age. Data are shown for a total of 223 CHEU and CHU combined**.** Black square = CHEU, hollow circle = CHU. Line of best fit is from a linear regression model with shading representing the 95% confidence interval. A) Total skinfold thickness. B) Lean mass index. C) Knee-heel length. D) MUAC Z-score.

Initial plots used Least-Squares linear regression to investigate associations across the whole cohort (Fig 2). At 2 years of age, mean (SD) LAZ was −1.47 (1.07) and 36/223 (16%) were stunted. Fig 2 shows the associations between body composition parameters and LAZ in the body composition substudy. Total skinfolds were not significantly associated with unit increases in LAZ (0.35, 95%CI −0.15, 0.85, p = 0.17; Fig 2A). By contrast, the lean mass index increased by 0.23 Ohms^-1^ per unit rise in LAZ (95%CI 0.06, 0.38, p = 0.005; Fig 2B). This shows that even after correcting for the effect of height on lean mass, the lean mass index shows a small but significant relationship with height. Each unit increase in LAZ was associated with an increase of 0.92 cm in knee-heel length (95%CI 0.83, 1.01, p < 0.001; Fig 2C) and 0.36 Z-score increase in MUAC (95%CI 0.27, 0.46, p < 0.001). Every 1 Kg increase in birthweight was associated with a 0.70 increase in LAZ at 2 years (95% CI 0.37, 1.03, p < 0.001).

### Body composition of CHEU vs. CHU

Table 2 shows the absolute body composition values among CHEU and CHU. Table 3 compares body composition of CHEU versus CHU, using four different models. In the unadjusted model, CHEU had lower LAZ, WAZ, MUAC and HCZ compared to CHU, as previously reported in the whole cohort [35]. In addition, calf circumference, knee-heel length, peripheral skinfolds, total skinfolds and impedance index were all reduced in CHEU versus CHU. There was no significant difference between groups for lean mass index or phase angle. When adjusted for trial arm only (model 2), findings were similar. When adjusted for trial arm, study nurse, exact age, sex and date of visit (model 3), HCZ was no longer significantly different between groups but other findings remained similar. Model 4 included further adjustments for maternal height, maternal education, maternal MUAC during pregnancy, religion, maternal Edinburgh Postnatal Depression scale, wealth index quintile, and household size. In this model, there was only weak evidence that HCZ and calf skinfolds were significantly different between groups, while other inferences remained similar.

**Table 2:** Body composition values of CHU and CHEU at 2 years of age.

Parameter	CHEU		CHU		P-value
	**n**	**Mean (SD)**	**n**	**Mean (SD)**	
**Female N, %**	19 (53%)	–	98 (52%)	–	0.57
**Age, months**	36	24.4 (0.5)	187	24.4 (0.4)	0.49
**Length, cm**	36	80.3 (3.1)	186	82.5 (3.4)	<0.001
**LAZ**	36	−2.1 (1.0)	186	−1.3 (1.0)	<0.001
**Weight, Kg**	36	10.2 (1.1)	186	11.0 (1.3)	<0.001
**WAZ**	36	−1.4 (1.0)	186	−0.7 (1.0)	<0.001
**Head circumference, cm**	36	47.6 (1.6)	186	48.1 (1.5)	0.10
**HCZ**	36	−0.1 (1.1)	186	0.2 (1.1)	0.04
**MUAC, cm**	36	14.7 (0.9)	187	15.3 (1.0)	<0.001
**MUAC Z-score**	36	−0.4 (0.8)	186	0.2 (0.8)	<0.001
**Calf circumference, cm**	35	17.8 (1.3)	184	18.7 (1.2)	<0.001
**Knee-heel length, cm**	35	22.8 (1.2)	187	23.4 (1.2)	<0.001
Impedance Index *	34	7.4 (1.1)	162	7.9 (1.2)	<0.001
**Lean mass index, Ohms**^**-1**^ **	34	11.4 (1.3)	163	11.5 (1.2)	0.68
**Phase angle, degrees**	34	3.8 (0.9)	163	4.0 (0.8)	0.32
**Subscapular SFT, mm**	35	7.1 (1.4)	186	7.6 (1.5)	0.06
**Subscapular Z-score**	35	−0.6 (1.1)	186	−0.2 (1.0)	0.07
**Triceps SFT, mm**	35	8.2 (1.7)	186	8.9 (1.7)	0.02
**Triceps SFT Z-score**	35	0.1 (1.0)	186	0.5 (0.9)	0.02
**Calf SFT, mm**	35	9.3 (1.5)	185	10.0 (1.5)	0.01
Peripheral SFT***, mm	35	17.5 (2.9)	185	18.9 (2.9)	0.01
**Total SFT, mm**	35	24.6 (4.0)	185	26.5 (4.1)	0.01

LAZ: Length-for-age Z-score, WAZ: Weight-for-age Z-score, HCZ: Head circumference for age Z-score, MUAC: Mid upper arm circumference, LMI: Lean mass index, SFT: skinfold thickness. * Impedance index used as a proxy for absolute lean mass. ** 1/Z is used as a proxy for the height-adjusted lean mass index *** Peripheral skinfold thickness used the sum of triceps and calf skinfolds.

**Table 3:** Comparison of body composition of CHEU with CHU at 2 years of age.

Statistical model	Model 1: Unadjusted absolute difference (95%CI) (CHEU versus CHUU)		Model 2: Adjusted absolute difference (CHEU versus CHUU)		Model 3: Adjusted absolute difference (CHEU versus CHUU)		Model 4: Adjusted absolute difference (CHEU versus CHUU)	
Parameter	Coeff (95% CI)	P	Coeff (95% CI)	P	Coeff (95% CI)	P	Coeff (95% CI)	P
**Length, cm**	**−2.16 (−3.22, −1.10)**	**<0.001**	**−2.17 (−3.21, −1.14)**	**<0.001**	**−2.44 (−3.39, −1.50)**	**<0.001**	**−2.15 (−3.38, −0.91)**	**<0.001**
**LAZ**	**−0.78 (−1.13, −0.43)**	**<0.001**	**−0.79 (−1.14, −0.43)**	**<0.001**	**−0.84 (−1.16, −0.51)**	**<0.001**	**−0.79 (−1.14, −0.43)**	**<0.001**
**Weight, Kg**	**−0.85 (−1.22, −0.48)**	**<0.001**	**−0.89 (−1.24, −0.54)**	**<0.001**	**−0.98 (−1.34, −0.62)**	**<0.001**	**−0.87 (−1.31, −0.42)**	**<0.001**
**WAZ**	**−0.72 (−1.04, −0.40)**	**<0.001**	**−0.79 (−1.12, −0.45)**	**<0.001**	**−0.85 (−1.16, −0.54)**	**<0.001**	**−0.79 (−1.12, −0.45)**	**<0.001**
**Head circumference, cm**	−0.44 (−0.96, 0.08)	0.10	−0.46 (−0.96, 0.03)	0.07	−0.47 (−0.99, 0.04)	0.07	−0.44 (−1.01, 0.14)	0.13
**HCZ**	**−0.38 (−0.74, −0.01)**	**0.04**	**−0.39 (−0.74, −0.04)**	**0.03**	−0.34 (−0.71, 0.03)	0.07	−0.31 (−0.73, 0.10)	0.14
**MUAC, cm**	**−0.63 (−0.95, −0.32)**	**<0.001**	**−0.63 (−0.95, −0.30)**	**<0.001**	**−0.69 (−1.06, −0.33)**	**<0.001**	**−0.68 (−1.07, −0.29)**	**0.001**
**MUAC Z-score**	**−0.55 (−0.82, −0.28)**	**<0.001**	**−0.55 (−0.83, −0.26)**	**<0.001**	**−0.58 (−0.89, −0.27)**	**<0.001**	**−0.58 (−0.91, −0.25)**	**0.001**
**Calf circumference, cm**	**−0.82 (−1.24, −0.40)**	**0.02**	**−0.84 (−1.25, −0.43)**	**<0.001**	**−0.95 (−1.35, −0.54)**	**<0.001**	**−0.84 (−1.25, −0.43)**	**<0.001**
**Knee heel length, cm**	**−0.68 (−1.06, −0.31)**	**<0.001**	**−0.71 (−1.09, −0.34)**	**<0.001**	**−0.77 (−1.13, −0.41)**	**<0.001**	**−0.71 (−1.09, −0.34)**	**<0.001**
Impedance Index *	**−0.49 (−0.80, −0.17)**	**<0.001**	**−0.51 (−0.86, −0.17)**	**<0.001**	**−0.56 (−0.87, −0.24)**	**<0.001**	**−0.49 (−0.87, −0.11)**	**0.01**
Lean mass index, Ohms^-1^ **	−0.09 (−0.53, 0.35)	0.68	−0.12 (−0.57, 0.34)	0.62	−0.12 (−0.54, 0.30)	0.57	−0.14 (−0.54, 0.25)	0.48
Phase angle, degrees	−0.17 (−0.50, 0.16)	0.32	−0.16 (−0.48, 0.15)	0.31	−0.09 (−0.38, 0.19)	0.52	−0.01 (−0.27, 0.25)	0.94
**Subscapular SFT, mm**	−0.54 (−1.10, 0.03)	0.06	−0.56 (−1.12, −0.01)	0.05	**−0.59 (−1.16, −0.02)**	**0.04**	−0.64 (−1.30, 0.02)	0.06
**Subscapular Z-score**	−0.39 (−0.82, 0.03)	0.07	−0.41 (−0.83, 0.01)	0.06	**−0.43 (−0.86, −0.01)**	**0.05**	**−0.49 (−0.97, −0.02)**	**0.04**
**Triceps SFT, mm**	**−0.69 (−1.27, −0.12)**	**0.02**	**−0.69 (−1.26, −0.12)**	**0.02**	**−0.82 (−1.41, −0.24)**	**0.006**	**−0.87 (−1.54, −0.20)**	**0.01**
**Triceps SFT Z-score**	**−0.41 (−0.76, −0.06)**	**0.02**	**−0.40 (−0.75, −0.06)**	**0.02**	**−0.48 (−0.83, −0.13)**	**0.008**	**−0.52 (−0.90, −0.13)**	**0.01**
**Calf SFT, mm**	**−0.67 (−1.18, −0.16)**	**0.01**	**−0.64 (−1.15, −0.12)**	**0.02**	**−0.63 (−1.20, −0.05)**	**0.03**	−0.59 (−1.25, 0.07)	0.08
Peripheral SFT***, mm	**−1.34 (−2.29, −0.38)**	**0.01**	**−1.30 (−2.27, −0.33)**	**0.01**	**−1.44 (−2.52, −0.35)**	**0.009**	**−1.44 (−2.70, −0.19)**	**0.02**
**Total SFT, mm**	**−1.87 (−3.33, −0.42)**	**0.01**	**−1.86 (−3.32, −0.39)**	**0.01**	**−2.03 (−3.63, −0.42)**	**0.01**	**−2.09 (−3.96, −0.23)**	**0.03**

**Regression coefficients from generalised estimating equations (GEE)** LAZ: Length-for-age Z-score, WAZ: Weight-for-age Z-score, HCZ: Head circumference for age Z-score, MUAC: Mid upper arm circumference, LMI: Lean mass index, SFT: Skinfold thickness. Model 1 was unadjusted. Model 2 was adjusted for trial arm only. Model 3 was adjusted for arm, study nurse, child age, sex and date of visit. Model 4 was adjusted for arm, study nurse, child age, sex, date of visit, maternal height, maternal education, maternal MUAC, religion, maternal depression score (EPDS), wealth index quintile, and household size. Significant differences between CHEU and CHU are highlighted in bold. * Impedance index used as a proxy for absolute lean mass. ** 1/Z is used as a proxy for the height-adjusted lean mass index *** Peripheral skinfold thickness used the sum of triceps and calf skinfolds.

Taken together, CHEU at 2 years of age had evidence of poorer growth across a range of measures in all models. We did not find any evidence of interaction between HIV-exposure and stunting, LAZ or birthweight on any body composition outcome at 2 years (P > 0.10). Therefore, HIV exposure appears to be independently associated with outcomes of reduced growth, lean mass and skinfold thicknesses at age 2 years, and these findings cannot be attributed solely to restricted antenatal growth (i.e., lower birthweight) or poor postnatal linear growth (i.e., reduced LAZ) among CHEU.

## Discussion

CHEU are a vulnerable population with reduced growth, development and survival [1]. This study characterised CHEU body composition measurements in a cohort of rural Zimbabwean children to gain further insight into markers of long-term growth and health. We found significant differences in body composition at 2 years of age among CHEU compared to CHU, as summarised in supporting information, S2 Fig in S1 File. CHEU had evidence of reduced weight, height and mid-upper arm circumference, accompanied by lower lean mass and skinfold thicknesses, even after adjusting for multiple social and environmental confounders. There was no significant interaction with stunting, LAZ or birthweight. Together, these body composition measurements indicate a restricted pattern of growth which may contribute to the disparities in morbidity, mortality and neurodevelopment between CHEU and CHU. Collectively, our findings highlight how HIV exposure shapes the pattern and prioritisation of growth in the first thousand days, and indicate the need for longer-term follow-up of CHEU in sub-Saharan Africa to evaluate non-communicable disease (NCD) risks at older ages.

The effects of antenatal HIV exposure in this cohort were evident in early life: CHEU had lower birthweights and a two-fold higher proportion of stunting at 2 years (52.8%) compared to CHU (26.2%). However, the higher prevalence of stunting did not entirely explain the body composition differences between CHEU and CHU at 2 years of age, since we did not find any evidence of interaction between HIV exposure and stunting or LAZ on any growth or body composition outcome. We also found no evidence of interaction between HIV exposure and birthweight on any body composition outcomes at 2 years of age. Therefore, whilst CHEU were lighter at birth (Table 1), this lack of a statistically significant interaction on 2 year body composition outcomes suggested these differences were not solely due to antenatal growth restriction. Instead, there appears to be a distinct growth phenotype in CHEU, characterised by reduced lean and fat mass compared to CHU.

Lean mass as estimated by the impedance index was reduced in CHEU [11]. This is important as lean mass is associated with improved physical function [36], cognitive function [37] and reduced risk of chronic disease [38,39]. Low lean mass suggests a reduced ‘metabolic capacity’ for homeostasis of key physiological parameters including glucose and blood pressure [9]. With later exposure to a ‘metabolic load’ caused by obesogenic factors such as diet or reduced exercise, this causes greater susceptibility to chronic diseases such as diabetes and hypertension, in the so-called capacity-load model [9]. For example, survivors of severe malnutrition in childhood have been observed to have shorter height [40], as well as higher waist circumference and risk of metabolic syndrome [41]. Lean mass is itself an important marker of quality of growth in response to nutrition, particularly in the first 1000 days. After this, organ growth becomes increasingly canalised by growth hormone so it closely follows growth in linear stature [9,42,43]. In this cohort, the knee-heel length was reduced by approximately one-third of the overall length. This knee-heel length to total length ratio is similar to that previously measured among HIV-unexposed children in Denmark [44], suggesting that knee-heel length to total length proportions are conserved for both CHEU and CHU. Knee-heel length may provide further insights into the quality of growth. A study among older stunted children in Nepal has shown relative leg length or total height may be a proxy of organ (kidney) size and later cardio-metabolic risk factors such as higher blood pressure, so this may be an early indicator of future vulnerability to NCD risk [16].

CHEU had reduced skinfold thicknesses but this was not associated with height. Given the higher mortality among CHEU in this population [45], conserved skinfold thickness may confer a short-term survival benefit for children who are HIV-unexposed. These effects are also important for later health as the first thousand days is a crucial window for generating the trajectory of both adipocyte number and adipose depots [46,47]. Reduced skinfold thicknesses have been previously observed in CHEU in infancy; [48] we extend these findings in the current cohort by confirming that this difference is still evident at 2 years of age. Furthermore, the lack of any significant interaction of 2 year-old body composition with birthweight or LAZ suggests this is an ongoing postnatal restriction in subcutaneous fat. The reduction of subcutaneous adipose tissue in CHEU may reflect the metabolic cost of ongoing inflammation [49]. There is evidence of both innate and adaptive immune activation in CHEU, reflecting a metabolically costly pro-inflammatory milieu [1], as well as the presence of other inflammatory drivers such as cytomegalovirus [50]. Reduced fat mass may be partly mediated by leptin which controls lipoprotein functions, acute phase reactants, glucocorticoid metabolism, inflammation and immune function [51]. In one South African study, CHEU aged 7–11 years were 50% less likely to have excess fat compared to CHU, whilst breastfeeding was protective against obesity for both CHEU and CHU [52]. Trajectories of CHEU compared to CHU have shown some variability, with a study in Zambia suggesting poor growth was universal for both CHEU and CHU [53]. However, a South African study suggested CHEU have significantly worse growth trajectories [54], similar to the SHINE cohort [35]. It is also plausible that the antenatal exposures of CHEU to both HIV and ART may set a trajectory for body composition similar to children who are living with HIV. With HIV infection, patterns of abnormal fat distribution vary from peripheral fat wasting alone or in combination with central fat accumulation [55]. For children living with HIV, lipoatrophy has been observed with reduced skinfold thicknesses, arm and leg circumferences [56], similar to that observed in this study. The intrauterine exposure to antiretrovirals also represent a plausible mechanism for the differences in body composition observed. Antiretrovirals have been associated with mitochondrial [57], neurologic and other toxicities [57] that may contribute to low birthweight [58,59], increased stunting in children [60] and therefore also altered body composition. For example, in an American cohort of CHEU, reduced skinfolds were postulated to be partly due to mitochondrial changes induced by antenatal HIV or ART exposures [61]. Future work characterising these biological mechanisms would be valuable, including measuring mitochondrial function and stability within CHEU [62] as well as indirect calorimetry to calculate resting energy expenditure, which is higher in children living with HIV (CLWH) [63]. Inflammatory and growth markers perturbed in early life among CHEU, such as CRP, IGF-1 [64] and leptin/adiponectin [65], should be monitored alongside other body composition drivers such as insulin sensitivity [66] and bone mineral density, which is also perturbed in CLWH [67]. Further insights from breast milk [68], gut inflammation, microbiota [69] and metabolomics [70] may also illuminate the drivers for these body composition differences. There is some evidence that differences in CHEU body composition may reduce by school-age [71], but long-term CHEU outcomes remain poorly understood.

This study provides additional information on early-life growth among CHEU and CHU, suggesting reduced lean mass in addition to reduced skinfold thickness. This supports the reduced length and skinfolds previously observed in Uganda at 12 months, which similarly had a high burden of childhood stunting [20]. We used a range of standardized techniques to characterise the quality of growth, and deployed rigorous HIV testing algorithms to correctly identify CHEU and CHU. We also demonstrated the techniques were fully portable and suitable for remote areas accessible by motorbike. This study also has some limitations. Gestational age was not available due to a lack of ultrasound dating scans. Therefore, it was not possible to explore if size-for-gestational-age was different by HIV status in this sample. The small number of CHEU (36) was also a limitation, particularly for the covariates included in adjusted models. The duration of breastfeeding was also not available, although reported breastfeeding rates >96% across all arms [7]. Children were recruited from a convenience sample in one part of the study area, so there may be some unintentional selection bias. However, baseline characteristics were broadly similar between those enrolled into the body composition sub‐study and the larger SHINE trial cohort. At baseline, only 40% of SHINE households met the minimum dietary diversity score, but by 12 months the IYCF arm had improved dietary diversity and iron-rich foods [7]. Of note, CHEU and CHU responded differently to the trial interventions, with CHEU randomised to IYCF showing average LAZ gains of 0·26 (95% CI 0·09–0·43) [26] compared to 0·16 (95% CI 0·08–0·23) among CHU [7]. Trial intervention arm was included in all adjusted analyses, but the study was not powered to examine the effect of the IYCF and WASH interventions on body composition. Evidence from the whole SHINE cohort suggested that the IYCF intervention improved growth across the cohort [7], particularly in CHEU [26]. Therefore it is plausible that postnatal factors such as nutritional supplements can improve growth, and mitigate stunting, as supported by a recent meta-analysis [72]. We extrapolate from this sub-study that improved linear growth could plausibly improve lean mass and knee-heel length but not skinfold thickness (given skinfold thickness was not associated with LAZ), but further studies of the effect of IYCF on body composition are needed. Finally, there were too few ART‐unexposed CHEU to explore the impact of ART (or specific regimens) on body composition or birthweight [59]. Most HIV‐positive mothers in SHINE received ART during the antenatal period, three‐quarters of whom were on a tenofovir‐based regimen. Furthermore, mothers were not randomized to ART use or regimen, so it would be difficult to ascertain whether any associations were due to specific ART.

## Conclusions

In conclusion the body composition of CHEU compared to CHU at 2 years of age shows clear reductions in subcutaneous fat and lean mass, which may illustrate both short-term vulnerabilities in health and mortality risk as well as long-term health outcomes. These differences were not wholly explained by the observed lower birthweight and reduced linear growth in CHEU, suggesting that other factors further restrict the quality of growth observed at 2 years, which may include HIV and ART exposure, or household socio-environmental factors. These results highlight the importance of the early-life environment in setting growth and body composition trajectories which are observable at 2 years of age. Future work exploring biological mechanisms includes further characterising the mitochondrial, gut, metabolic and inflammatory perturbations that may alter growth in CHEU. Longer term follow-up is required to monitor these trajectories further and to ascertain differences in NCD risk between CHEU and CHU. As the global population of CHEU grows and matures, there is clear need to adopt a life-course approach to identifying and supporting children at all stages [2], to promote long-term health, wellbeing and human capital.

## Supporting information

S1 File S1 Fig. Consort diagram for study. 212 clusters were randomized, 53 in each of the four trial arms. After randomization, one cluster was excluded as it was determined to be in an urban area, one cluster was excluded as the VHW covering it mainly had clients outside the study area, and one more was merged into a neighbouring cluster based on subsequent data on VHW coverage. Three new cluster designations were created due to anomalies in the original mapping; for two of these, the trial arm was clear—the third contained areas that were in two trial arms, and was assigned to the underrepresented arm, resulting in 53 clusters in each arm. All of this occurred before enrolment began. When enrolment was completed, however, there were two clusters (SOC, and WASH+IYCF) in which no women were enrolled, leaving a total of 210 clusters available for analysis. ^2^SOC=Standard of Care; IYCF = Infant and Young Child Feeding; WASH = Water and Sanitation/Hygiene. S2 Fig. Summary of overall findings. Skinfold thickness, bioimpedance and knee-heel length provided measures of fat mass, lean mass and growth proportions. Children born HIV-exposed uninfected (CHEU) had reduced growth, fat, lean and knee-heel length compared to children born unexposed to HIV (CHU). S3 Fig. Comparison of knemometer types. Bland-Altman plot comparing custom-built and adapted knemometers, showing mean difference and 95% limits of agreement. **S1 Table. Baseline characteristics of children enrolled** or not enrolled into the body composition substudy.
(DOCX)

S1 Data Anonymised dataset of the body composition substudy. (DTA)

## References

[1] Evans C, Jones CE, Prendergast AJ. HIV-exposed, uninfected infants: new global challenges in the era of paediatric HIV elimination. Lancet Infect Dis. 2016;16(6):e92–107. doi: 10.1016/S1473-3099(16)00055-4 27049574

[2] Ramokolo V, Goga AE, Slogrove AL, Powis KM. Unmasking the vulnerabilities of uninfected children exposed to HIV. BMJ. 2019;366:l4479. doi: 10.1136/bmj.l4479 31383646 PMC6894435

[3] Evans C, Humphrey JH, Ntozini R, Prendergast AJ. HIV-exposed uninfected infants in Zimbabwe: insights into health outcomes in the pre-antiretroviral therapy era. Front Immunol. 2016;7. doi: 10.3389/fimmu.2016.00190 PMC4893498 27375613

[4] Jumare J, Datong P, Osawe S, Okolo F, Mohammed S, Inyang B, et al. Compromised Growth Among HIV-exposed Uninfected Compared With Unexposed Children in Nigeria. Pediatr Infect Dis J. 2019;38(3):280–6. doi: 10.1097/INF.0000000000002238 30418356

[5] Rosala-Hallas A, Bartlett JW, Filteau S. Growth of HIV-exposed uninfected, compared with HIV-unexposed, Zambian children: a longitudinal analysis from infancy to school age. BMC Pediatr. 2017;17(1):80. doi: 10.1186/s12887-017-0828-6 28302082 PMC5356250

[6] le Roux SM, Abrams EJ, Donald KA, Brittain K, Phillips TK, Nguyen KK, et al. Growth trajectories of breastfed HIV-exposed uninfected and HIV-unexposed children under conditions of universal maternal antiretroviral therapy: a prospective study. Lancet Child Adolesc Health. 2019;3(4):234–44. doi: 10.1016/S2352-4642(19)30007-0 30773459

[7] Humphrey JH, Mbuya MNN, Ntozini R, Moulton LH, Stoltzfus RJ, Tavengwa NV, et al. Independent and combined effects of improved water, sanitation, and hygiene, and improved complementary feeding, on child stunting and anaemia in rural Zimbabwe: a cluster-randomised trial. Lancet Glob Health. 2019;7(1):e132–47. doi: 10.1016/S2214-109X(18)30374-7 30554749 PMC6293965

[8] Martorell R. Improved nutrition in the first 1000 days and adult human capital and health. Am J Hum Biol. 2017;29(2). doi: 10.1002/ajhb.22952 PMC5761352 28117514

[9] Wells JCK. The capacity-load model of non-communicable disease risk: understanding the effects of child malnutrition, ethnicity and the social determinants of health. Eur J Clin Nutr. 2018;72(5):688–97. doi: 10.1038/s41430-018-0142-x 29748656

[10] Wells JCK, Devakumar D, Manandhar DS, Saville N, Chaube SS, Costello A, et al. Associations of stunting at 2 years with body composition and blood pressure at 8 years of age: longitudinal cohort analysis from lowland Nepal. Eur J Clin Nutr. 2019;73(2):302–10. doi: 10.1038/s41430-018-0291-y 30154534 PMC6368558

[11] Wells JCK. Body composition of children with moderate and severe undernutrition and after treatment: a narrative review. BMC Med. 2019;17(1):215. doi: 10.1186/s12916-019-1465-8 31767002 PMC6878632

[12] Bartz S, Mody A, Hornik C, Bain J, Muehlbauer M, Kiyimba T, et al. Severe acute malnutrition in childhood: hormonal and metabolic status at presentation, response to treatment, and predictors of mortality. J Clin Endocrinol Metab. 2014;99(6):2128–37. doi: 10.1210/jc.2013-4018 24606092 PMC4037734

[13] Gabrielsson BG, Johansson JM, Lönn M, Jernås M, Olbers T, Peltonen M, et al. High expression of complement components in omental adipose tissue in obese men. Obes Res. 2003;11(6):699–708. doi: 10.1038/oby.2003.100 12805391

[14] Chait A, den Hartigh LJ. Adipose Tissue Distribution, Inflammation and Its Metabolic Consequences, Including Diabetes and Cardiovascular Disease. Front Cardiovasc Med. 2020;7:22. doi: 10.3389/fcvm.2020.00022 32158768 PMC7052117

[15] Wells JCK, Cortina-Borja M. Different associations of subscapular and triceps skinfold thicknesses with pathogen load: an ecogeographical analysis. Am J Hum Biol. 2013;25(5):594–605. doi: 10.1002/ajhb.22418 23913438

[16] Wells JC, Devakumar D, Grijalva-Eternod CS, Manandhar DS, Costello A, Osrin D. Blood pressure and the capacity-load model in 8-year-old children from Nepal: testing the contributions of kidney size and intergenerational effects. Am J Hum Biol. 2016;28(4):555–65. doi: 10.1002/ajhb.22829 26848931 PMC7611548

[17] Wells JCK. The programming effects of early growth. Early Hum Dev. 2007;83(12):743–8. doi: 10.1016/j.earlhumdev.2007.09.002 17904771

[18] Abera M, Tesfaye M, Admassu B, Hanlon C, Ritz C, Wibaek R, et al. Body composition during early infancy and developmental progression from 1 to 5 years of age: the Infant Anthropometry and Body Composition (iABC) cohort study among Ethiopian children. Br J Nutr. 2018;119(11):1263–73. doi: 10.1017/S000711451800082X 29770755

[19] Rickman RR, Lane CE, Collins SM, Miller JD, Onono M, Wekesa P, et al. Body Composition Trajectories During the First 23 Months of Life Differ by HIV Exposure Among Infants in Western Kenya: A Prospective Study. J Nutr. 2023;153(1):331–9. doi: 10.1016/j.tjnut.2022.11.010 36913469 PMC10196592

[20] Lane CE, Widen EM, Collins SM, Young SL. HIV-Exposed, Uninfected Infants in Uganda Experience Poorer Growth and Body Composition Trajectories than HIV-Unexposed Infants. J Acquir Immune Defic Syndr. 2020;85(2):138–47. doi: 10.1097/QAI.0000000000002428 32604132 PMC7492413

[21] Neri D, Somarriba GA, Schaefer NN, Chaparro AI, Scott GB, Lopez Mitnik G, et al. Growth and body composition of uninfected children exposed to human immunodeficiency virus: comparison with a contemporary cohort and United States National Standards. J Pediatr. 2013;163(1):249-54.e1-2. doi: 10.1016/j.jpeds.2012.12.034 23360565 PMC3641163

[22] Strydom K, Nel DG, Dhansay MA, Van Niekerk E. The effect of maternal HIV status and treatment duration on body composition of HIV-exposed and HIV-unexposed preterm, very and extremely low-birthweight infants. Paediatr Int Child Health. 2018;38(3):163–74. doi: 10.1080/20469047.2018.1466481 29790827

[23] Nicholson L, Chisenga M, Siame J, Kasonka L, Filteau S. Growth and health outcomes at school age in HIV-exposed, uninfected Zambian children: follow-up of two cohorts studied in infancy. BMC Pediatr. 2015;15:66. doi: 10.1186/s12887-015-0386-8 26048411 PMC4458018

[24] Ntozini R, Chandna J, Evans C, Chasekwa B, Majo FD, Kandawasvika G, et al. Early child development in children who are HIV-exposed uninfected compared to children who are HIV-unexposed: observational sub-study of a cluster-randomized trial in rural Zimbabwe. J Int AIDS Soc. 2020;23(5):e25456. doi: 10.1002/jia2.25456 32386127 PMC7318086

[25] The Sanitation Hygiene Infant Nutrition Efficacy Trial Team. The Sanitation Hygiene Infant Nutrition Efficacy (SHINE) Trial: Rationale, Design, and Methods. Clin Infect Dis. 2015;61(suppl 7):S685–702. doi: 10.1093/cid/civ844 PMC4657589 26602296

[26] Prendergast AJ, Chasekwa B, Evans C, Mutasa K, Mbuya MNN, Stoltzfus RJ, et al. Independent and combined effects of improved water, sanitation, and hygiene, and improved complementary feeding, on stunting and anaemia among HIV-exposed children in rural Zimbabwe: a cluster-randomised controlled trial. Lancet Child Adolesc Health. 2019;3(2):77–90. doi: 10.1016/S2352-4642(18)30340-7 30573417 PMC6472652

[27] Norman K, Smoliner C, Kilbert A, Valentini L, Lochs H, Pirlich M. Disease-related malnutrition but not underweight by BMI is reflected by disturbed electric tissue properties in the bioelectrical impedance vector analysis. Br J Nutr. 2008;100(3):590–5. doi: 10.1017/S0007114508911545 18234142

[28] Kumar S, Dutt A, Hemraj S, Bhat S, Manipadybhima B. Phase Angle Measurement in Healthy Human Subjects through Bio-Impedance Analysis. Iran J Basic Med Sci. 2012;15(6):1180–4. 23653848 PMC3646229

[29] Wells JCK, Williams JE, Fewtrell M, Singhal A, Lucas A, Cole TJ. A simplified approach to analysing bio-electrical impedance data in epidemiological surveys. Int J Obes (Lond). 2007;31(3):507–14. doi: 10.1038/sj.ijo.0803441 16894361

[30] Parker L, Reilly JJ, Slater C, Wells JCK, Pitsiladis Y. Validity of six field and laboratory methods for measurement of body composition in boys. Obes Res. 2003;11(7):852–8. doi: 10.1038/oby.2003.117 12855754

[31] Nightingale CM, Rudnicka AR, Owen CG, Donin AS, Newton SL, Furness CA, et al. Are ethnic and gender specific equations needed to derive fat free mass from bioelectrical impedance in children of South asian, black african-Caribbean and white European origin? Results of the assessment of body composition in children study. PLoS One. 2013;8(10):e76426. doi: 10.1371/journal.pone.0076426 24204625 PMC3799736

[32] Norman K, Stobäus N, Pirlich M, Bosy-Westphal A. Bioelectrical phase angle and impedance vector analysis--clinical relevance and applicability of impedance parameters. Clin Nutr. 2012;31(6):854–61. doi: 10.1016/j.clnu.2012.05.008 22698802

[33] Gladstone MJ, Chandna J, Kandawasvika G, Ntozini R, Majo FD, Tavengwa NV, et al. Independent and combined effects of improved water, sanitation, and hygiene (WASH) and improved complementary feeding on early neurodevelopment among children born to HIV-negative mothers in rural Zimbabwe: Substudy of a cluster-randomized trial. PLoS Med. 2019;16(3):e1002766. doi: 10.1371/journal.pmed.1002766 30897095 PMC6428259

[34] Sanitation Hygiene Infant Nutrition Efficacy (SHINE) Trial Team, Humphrey JH, Jones AD, Manges A, Mangwadu G, Maluccio JA, et al. The Sanitation Hygiene Infant Nutrition Efficacy (SHINE) Trial: Rationale, Design, and Methods. Clin Infect Dis. 2015;61 Suppl 7(Suppl 7):S685-702. doi: 10.1093/cid/civ844 26602296 PMC4657589

[35] Evans C, Chasekwa B, Ntozini R, Majo FD, Mutasa K, Tavengwa N, et al. Mortality, Human Immunodeficiency Virus (HIV) Transmission, and Growth in Children Exposed to HIV in Rural Zimbabwe. Clin Infect Dis. 2021;72(4):586–94. doi: 10.1093/cid/ciaa076 31974572 PMC7884806

[36] Schumann M, Küüsmaa M, Newton RU, Sirparanta A-I, Syväoja H, Häkkinen A, et al. Fitness and lean mass increases during combined training independent of loading order. Med Sci Sports Exerc. 2014;46(9):1758–68. doi: 10.1249/MSS.0000000000000303 24518195

[37] Abera M, Tesfaye M, Hanlon C, Admassu B, Girma T, Wells JC, et al. Body Composition during Early Infancy and Mental Health Outcomes at 5 Years of Age: A Prospective Cohort Study of Ethiopian Children. J Pediatr. 2018;200:225–31. doi: 10.1016/j.jpeds.2018.04.055 30060887

[38] Wells JCK. The capacity-load model of non-communicable disease risk: understanding the effects of child malnutrition, ethnicity and the social determinants of health. Eur J Clin Nutr. 2018;72(5):688–97. doi: 10.1038/s41430-018-0142-x 29748656

[39] Ruiz JR, Castro-Piñero J, Artero EG, Ortega FB, Sjöström M, Suni J, et al. Predictive validity of health-related fitness in youth: a systematic review. Br J Sports Med. 2009;43(12):909–23. doi: 10.1136/bjsm.2008.056499 19158130

[40] Mwene-Batu P, Wells J, Maheshe G, Hermans MP, Kalumuna E, Ngaboyeka G. Body composition of adults with a history of severe acute malnutrition during childhood using the deuterium dilution method in eastern DR Congo: the Lwiro Cohort Study. Am J Clin Nutr. 2021. doi: 10.1093/ajcn/nqab293 34582550 PMC8634579

[41] Mwene-Batu P, Bisimwa G, Ngaboyeka G, Dramaix M, Macq J, Hermans MP, et al. Severe acute malnutrition in childhood, chronic diseases, and human capital in adulthood in the Democratic Republic of Congo: the Lwiro Cohort Study. Am J Clin Nutr. 2021;114(1):70–9. doi: 10.1093/ajcn/nqab034 33826712 PMC8246611

[42] Coppoletta JM, Wolbach SB. Body Length and Organ Weights of Infants and Children: A Study of the Body Length and Normal Weights of the More Important Vital Organs of the Body between Birth and Twelve Years of Age. Am J Pathol. 1933;9(1):55–70. 19970058 PMC2062747

[43] de la Grandmaison GL, Clairand I, Durigon M. Organ weight in 684 adult autopsies: new tables for a Caucasoid population. Forensic Sci Int. 2001;119(2):149–54. doi: 10.1016/s0379-0738(00)00401-1 11376980

[44] Michaelsen KF. Short-term measurements of linear growth using knemometry. J Pediatr Endocrinol. 1994;7(2):147–54. doi: 10.1515/jpem.1994.7.2.147 8061760

[45] Evans C, Chasekwa B, Ntozini R, Majo FD, Mutasa K, Tavengwa N, et al. Surviving and thriving? Outcomes of HIV-exposed children in rural Zimbabwe. Conference on Retroviruses and Opportunistic Infections (CROI); 4th–7th March, 2019; Seattle (WA). 2019.

[46] Spalding KL, Arner E, Westermark PO, Bernard S, Buchholz BA, Bergmann O, et al. Dynamics of fat cell turnover in humans. Nature. 2008;453(7196):783–7. doi: 10.1038/nature06902 18454136

[47] Mameli C, Mazzantini S, Zuccotti GV. Nutrition in the First 1000 Days: The Origin of Childhood Obesity. Int J Environ Res Public Health. 2016;13(9):838. doi: 10.3390/ijerph13090838 27563917 PMC5036671

[48] Strydom K, Nel DG, Dhansay MA, Van Niekerk E. The effect of maternal HIV status and treatment duration on body composition of HIV-exposed and HIV-unexposed preterm, very and extremely low-birthweight infants. Paediatr Int Child Health. 2018;38(3):163–74. doi: 10.1080/20469047.2018.1466481 29790827

[49] Wells JCK. Ethnic variability in adiposity and cardiovascular risk: the variable disease selection hypothesis. Int J Epidemiol. 2009;38(1):63–71. doi: 10.1093/ije/dyn183 18820320

[50] Evans C, Chasekwa B, Rukobo S, Govha M, Mutasa K, Ntozini R, et al. CMV acquisition and inflammation in HIV-exposed uninfected Zimbabwean infants. J Infect Dis. 2016;jiw630. doi: 10.1093/infdis/jiw630 PMC5388301 28011912

[51] Härle P, Straub RH. Leptin is a link between adipose tissue and inflammation. Ann N Y Acad Sci. 2006;1069:454–62. doi: 10.1196/annals.1351.044 16855173

[52] Houle B, Rochat TJ, Newell M-L, Stein A, Bland RM. Breastfeeding, HIV exposure, childhood obesity, and prehypertension: A South African cohort study. PLoS Med. 2019;16(8):e1002889. doi: 10.1371/journal.pmed.1002889 31454346 PMC6711496

[53] Chilyabanyama ON, Chilengi R, Laban NM, Chirwa M, Simunyandi M, Hatyoka LM, et al. Comparing growth velocity of HIV exposed and non-exposed infants: An observational study of infants enrolled in a randomized control trial in Zambia. PLoS One. 2021;16(8):e0256443. doi: 10.1371/journal.pone.0256443 34424916 PMC8382174

[54] Pillay L, Moodley D, Emel LM, Nkwanyana NM, Naidoo K. Growth patterns and clinical outcomes in association with breastfeeding duration in HIV exposed and unexposed infants: a cohort study in KwaZulu Natal, South Africa. BMC Pediatr. 2021;21(1):183. doi: 10.1186/s12887-021-02662-8 33874900 PMC8054353

[55] Carr A, Cooper DA. Adverse effects of antiretroviral therapy. Lancet. 2000;356(9239):1423–30. doi: 10.1016/S0140-6736(00)02854-3 11052597

[56] Hartman K, Verweel G, de Groot R, Hartwig NG. Detection of lipoatrophy in human immunodeficiency virus-1-infected children treated with highly active antiretroviral therapy. Pediatr Infect Dis J. 2006;25(5):427–31. doi: 10.1097/01.inf.0000215003.32256.aa 16645507

[57] Williams PL, Hazra R, Van Dyke RB, Yildirim C, Crain MJ, Seage GR 3rd, et al. Antiretroviral exposure during pregnancy and adverse outcomes in HIV-exposed uninfected infants and children using a trigger-based design. AIDS. 2016;30(1):133–44. doi: 10.1097/QAD.0000000000000916 26731758 PMC4704129

[58] Delicio AM, Lajos GJ, Amaral E, Cavichiolli F, Polydoro M, Milanez H. Adverse effects in children exposed to maternal HIV and antiretroviral therapy during pregnancy in Brazil: a cohort study. Reprod Health. 2018;15(1):76. doi: 10.1186/s12978-018-0513-8 29747664 PMC5946413

[59] Njom Nlend AE, Nga Motazé A, Moyo Tetang S, Zeudja C, Ngantcha M, Tejiokem M. Preterm Birth and Low Birth Weight after In Utero Exposure to Antiretrovirals Initiated during Pregnancy in Yaoundé, Cameroon. PLoS One. 2016;11(3):e0150565. doi: 10.1371/journal.pone.0150565 26999744 PMC4801361

[60] Wedderburn CJ, Yeung S, Rehman AM, Stadler JAM, Nhapi RT, Barnett W, et al. Neurodevelopment of HIV-exposed uninfected children in South Africa: outcomes from an observational birth cohort study. Lancet Child Adolesc Health. 2019;3(11):803–13. doi: 10.1016/S2352-4642(19)30250-0 31515160 PMC6876655

[61] Neri D, Somarriba GA, Schaefer NN, Chaparro AI, Scott GB, Lopez Mitnik G, et al. Growth and body composition of uninfected children exposed to human immunodeficiency virus: comparison with a contemporary cohort and United States National Standards. J Pediatr. 2013;163(1):249–54.e1-2. doi: 10.1016/j.jpeds.2012.12.034 23360565 PMC3641163

[62] Monnin A, Desquiret-Dumas V, Méda N, Goudenège D, Bris C, Kankasa C, et al. Mitochondrial DNA Instability Is Common in HIV-Exposed Uninfected Newborns. J Clin Med. 2021;10(11):2399. doi: 10.3390/jcm10112399 34071681 PMC8197798

[63] Franco-Oliva A, Pinzón-Navarro BA, Martínez-Soto-Holguín MC, León-Lara X, Ordoñez-Ortega J, Pardo-Gutiérrez AL, et al. High resting energy expenditure, less fat-free mass, and less muscle strength in HIV-infected children: a matched, cross-sectional study. Front Nutr. 2023;10:1220013. doi: 10.3389/fnut.2023.1220013 37799766 PMC10548389

[64] Evans C, Chasekwa B, Rukobo S, Govha M, Mutasa K, Ntozini R, et al. Inflammation, cytomegalovirus and the growth hormone axis in HIV-exposed uninfected Zimbabwean infants. AIDS. 2020;34(14):2045–50. doi: 10.1097/QAD.0000000000002646 32773472 PMC7610753

[65] Onubogu CU, Ugochukwu EF, Okpara HC. Cord blood leptin levels and anthropometric indices in virally suppressed HIV-positive and HIV-negative mother-singleton newborn pairs: a comparative analysis. Niger J Clin Pract. 2025;28(1):8–18. doi: 10.4103/njcp.njcp_605_24 40326931

[66] Jao J, Sun S, Bonner LB, Legbedze J, Mmasa KN, Makhema J, et al. Lower Insulin Sensitivity in Newborns With In Utero HIV and Antiretroviral Exposure Who Are Uninfected in Botswana. J Infect Dis. 2022;226(11):2002–9. doi: 10.1093/infdis/jiac416 36240387 PMC10205604

[67] Mukwasi-Kahari C, Rehman AM, Ó Breasail M, Rukuni R, Madanhire T, Chipanga J, et al. Impaired Bone Architecture in Peripubertal Children With HIV, Despite Treatment With Antiretroviral Therapy: A Cross-Sectional Study From Zimbabwe. J Bone Miner Res. 2023;38(2):248–60. doi: 10.1002/jbmr.4752 36426511 PMC9996028

[68] Byrne A, Diener C, Brown BP, Maust BS, Feng C, Alinde BL, et al. Neonates exposed to HIV but uninfected exhibit an altered gut microbiota and inflammation associated with impaired breast milk antibody function. Microbiome. 2024;12(1):261. doi: 10.1186/s40168-024-01973-z 39707483 PMC11662858

[69] Jackson CL, Frank DN, Robertson CE, Ir D, Kofonow JM, Montlha MP, et al. Evolution of the Gut Microbiome in HIV-Exposed Uninfected and Unexposed Infants during the First Year of Life. mBio. 2022;13(5):e0122922. doi: 10.1128/mbio.01229-22 36073815 PMC9600264

[70] du Toit LDV, Mason S, van Reenen M, Rossouw TM, Louw R. Metabolic Alterations in Mothers Living with HIV and Their HIV-Exposed, Uninfected Infants. Viruses. 2024;16(2):313. doi: 10.3390/v16020313 38400088 PMC10892778

[71] Piper JD, Mazhanga C, Mwapaura M, Mapako G, Mapurisa I, Mashedze T, et al. Growth, physical, and cognitive function in children who are born HIV-free: School-age follow-up of a cluster-randomised trial in rural Zimbabwe. PLoS Med. 2024;21(10):e1004347. doi: 10.1371/journal.pmed.1004347 39392862 PMC11498706

[72] Dewey KG, Stewart CP, Wessells KR, Prado EL, Arnold CD. Small-quantity lipid-based nutrient supplements for the prevention of child malnutrition and promotion of healthy development: overview of individual participant data meta-analysis and programmatic implications. Am J Clin Nutr. 2021;114(Suppl 1):3S–14S. doi: 10.1093/ajcn/nqab279 34590696 PMC8560310

