# Supplementary material for: Body composition of children born HIV-exposed uninfected in rural Zimbabwe
Source: PLoS One. 2026 Sep 11;21(9):e0319542. doi: 10.1371/journal.pone.0319542 (PMC13567772; doi:10.1371/journal.pone.0319542)
Supplement: Supplementary file 1 [file pone.0319542.s001.docx]

Supporting information

Table of Contents

[Supplementary Data	1](#_Toc189216539)

[Supplementary Figure 1: Consort Diagram	2](#_Toc189216540)

[Supplementary figure S2 Summary of overall findings	5](#_Toc189216541)

[Supplementary Table S1: Baseline characteristics	6](#_Toc189216542)

[Supplementary Methods	8](#_Toc189216543)

[HIV testing	8](#_Toc189216544)

[Skinfold Thicknesses	8](#_Toc189216545)

[Knee-heel length measurement	9](#_Toc189216546)

[Bioimpedance measurement	10](#_Toc189216547)

[Derivation of model 4	11](#_Toc189216548)

# Supplementary Data

## S1 Fig: Consort Diagram

**Supplementary Figure S1: CONSORT diagram of body composition substudy.** The selection of children across all arms of the SHINE trial is shown. Note that a maternal death on the CONSORT relates to a change of caregiver where the child was no longer able to be enrolled in the study.

**^1^**212 clusters were randomized, 53 in each of the four trial arms. After randomization, one cluster was excluded as it was determined to be in an urban area, one cluster was excluded as the VHW covering it mainly had clients outside the study area, and one more was merged into a neighbouring cluster based on subsequent data on VHW coverage. Three new cluster designations were created due to anomalies in the original mapping; for two of these, the trial arm was clear—the third contained areas that were in two trial arms, and was assigned to the underrepresented arm, resulting in 53 clusters in each arm. All of this occurred before enrolment began. When enrolment was completed, however, there were two clusters (SOC, and WASH+IYCF) in which no women were enrolled, leaving a total of 210 clusters available for analysis. ^2^SOC=Standard of Care; IYCF = Infant and Young Child Feeding; WASH = Water and Sanitation/Hygiene.

## S2 Fig: Summary of overall findings

Supplementary Figure S2: Summary of overall findings: Skinfold thickness, bioimpedance and knee-heel length provided measures of fat mass, lean mass and growth proportions. Children born HIV-exposed uninfected (CHEU) had reduced growth, fat, lean and knee-heel length.

## S1Table: Baseline characteristics

| **S1 Table: Baseline characteristics of children enrolled or not enrolled into the body composition substudy** | | | |
| --- | --- | --- | --- |
|  | Enrolled | Not enrolled | P value |
| Mothers, N | 221 | 4556 |  |
| Infants, N | 223 | 4620 |  |
| Trial Arm |  |  |  |
| SOC | 66/221 (29.9%) | 1087/4556 (23.9%) | 0.644 |
| IYCF | 47/221 (21.3%) | 1094/4556 (24.0%) |  |
| WASH | 41/221 (18.6%) | 1161/4556 (25.5%) |  |
| IYCF & WASH | 67/221 (30.3%) | 1214/4556 (26.7%) |  |
| Maternal characteristics |  |  |  |
| Mean years of schooling completed (SD) | 9.6 (1.9) | 9.5 (1.8) | 0.833 |
| Mean age (SD), years | 27.9 (6.6) | 26.1 (6.7) | <0.001 |
| Median parity (IQR) | 2 (1,3) | 2 (1,3) | 0.422 |
| Mean height (SD), cm | 159.7 (9.3) | 159.8 (8.4) | 0.935 |
| Mean mid-upper-arm circumference (SD), cm | 26.7 (3.1) | 26.4 (3.1) | 0.135 |
| Married | 201/209 (96.2%) | 4034/4242 (95.1%) | 0.643 |
| Employed | 18/215 (8.4%) | 361/4166 (8.7%) | 0.875 |
| Religion: |  |  |  |
| Apostolic | 95/211 (45.0%) | 2025/4275 (47.4%) | 0.018 |
| Other Christian | 107/211 (50.7%) | 1883/4257 (44.1%) |  |
| Other | 9/211 (4.3%) | 367/4257 (8.6%) |  |
| Mean Edinburgh postnatal depression scale (SD) | 0.9 (2.1) | 3.2 (4.4) | <0.001 |
| HIV disease severity and treatment (among HIV-positive mothers only) | | | |
| Mean CD4 count in pregnancy (SD), cells/µLc | 427 (199.5) | 487 (224.8) | 0.001 |
| Documented ART during pregnancy | 33/36 (91.7%) | 554/690 (80.3%) | 0.155 |
| Tenofovir disoproxil fumarate-based ART regimen | 24/33 (72.7%) | 367/554 (66.2%) | 0.439 |
| Zidovudine-based ART regimen | 4/33 (12.1%) | 112/554 (20.2%) | 0.263 |
| Other/unknown ART regimen | 5/33 (15.2%) | 75/554 (13.5%) | 0.81 |
| Documented co-trimoxazole prophylaxis during pregnancy | 19/36 (52.8%) | 383/690 (55.5%) | 0.04 |
| Household Characteristics |  |  |  |
| Median Number of Occupants [IQR] | 4 (3,5) | 5 (3,6) | 0.035 |
| Median Coping Strategies Index score [IQR] | 0 (0,3) | 1 (0,7) | <0.001 |
| Wealth Quintile: |  |  |  |
| Lowest | 35/216 (16.2%) | 837/4169 (20.1%) | 0.399 |
| Second | 45/216 (20.8%) | 832/4169 (20.0%) |  |
| Middle | 44/216 (20.4%) | 838/4169 (20.1%) |  |
| Fourth | 52/216 (24.1%) | 829/4169 (19.9%) |  |
| Highest | 40/216 (18.5%) | 833/4169 920.0%) |  |
| Electricity in home | 6/215 (2.8%) | 113/4165 (2.7%) | 0.954 |
| Improved latrine at household | 72/214 (33.6%) | 1296/4104 (31.6%) | 0.508 |
| Main source of household drinking water improved | 149/213 (70.0%) | 2578/4129 (62.4%) | 0.082 |
| Infant characteristics |  |  |  |
| Female sex | 115/223 (51.6%) | 2272/4594 (49.5%) | 0.513 |
| Mean birth weight (SD), kg | 3.1 (0.5) | 3.1 (0.5) | 0.996 |
| Birth weight <2.5kg | 16/220 (7.3%) | 409/4079 (10.0%) | 0.213 |
| Institutional delivery | 199/216 (92.1%) | 3604/4100 (87.9%) | 0.044 |
| Vaginal delivery | 199/220 (90.5%) | 3860/4166 (92.7%) | 0.029 |

# Supplementary Methods

The SHINE study was a cluster-randomized, community-based, 2x2 factorial trial testing the independent and combined effects of a water, sanitation and hygiene (WASH) intervention and an infant and young child feeding (IYCF) intervention on linear growth and hemoglobin concentration at 18 months of age. An initial pilot study was performed to check the acceptability and feasibility of the methods and 6-monthly standardisation exercises were performed. Of note, National PMTCT guidelines changed from World Health Organization (WHO) Option B (maternal ART from 14 gestational weeks until the end of breastfeeding) to Option B+ (lifelong ART for all pregnant and breastfeeding women) in November 2013. Viral loads were not measured.

Children were measured in the most convenient place in the household for the family, which also enabled them to be calm and reassured by their primary caregiver. Typically the children were measured in the kitchen or household yard.

## HIV testing

Mothers were tested in pregnancy using a rapid test algorithm (Alere Determine HIV-1/2 test, and, if positive, INSTI HIV-1/2 test [BioLytical Laboratories]) as previously reported(1). Women testing HIV-positive had CD4 counts measured (Alere Pima Analyser) and were referred to local clinics. Women were encouraged to initiate co-trimoxazole and ART, to exclusively breastfeed, and to attend clinic at 6 weeks postpartum for infant diagnosis and co-trimoxazole initiation. The first-line ART regimen in Zimbabwe was 2 nucleoside reverse transcriptase inhibitors (NRTIs) and a non-nucleoside reverse transcriptase inhibitor.

Children of HIV-positive mothers were offered longitudinal testing through 18 months of age; those testing positive were referred to local clinics for ART and were not included in this substudy. Children born to HIV-positive mothers who were confirmed HIV-negative at 18 months were classified as children HIV-exposed uninfected (CHEU). Children not tested at 18 months due to caregiver refusal, defaulted visits, or loss to follow-up were classified as HIV-unknown and were not included in this substudy. Children born from HIV-negative mothers were HIV-exposed (CHU) were defined as children born to HIV-negative mothers.

## Skinfold Thicknesses

The calipers (<https://holtain.co.uk/tw.php>) were explained to the caregiver. The calipers were calibrated in the field and the dial checked to be set at zero. A sample skinfold thickness (SFT) was often performed on the caregiver as a demonstration. The positioning for 2 year old skinfolds was standardised: The child was seated facing the caregiver and on her lap. The child had legs either side of mother and facing towards mother in the ‘bear hug’ position. In this position, the mother could hold the child’s arms to prevent movement and also use toys to provide a distraction. Typical toys used were hand puppets, clear plastic balls with further items inside, and local crisp packets. If the child was sleeping, all measurements could be conducted without waking them. Skinfolds were not measured over skin which was inflamed. If required, skinfolds were measured on the other side of the body.

White eyeliner pencils were used to mark the skin at the correct anatomical points, standardised to be on the left side. For Triceps SFT, the back of the arm, halfway between the acromion and olecranon was marked, using the same point as for the MUAC. For subscapular SFT, 1cm below the angle of the curve of the scapular surface anatomy was measured. For calf SFT, the back of the calf at its maximal circumference was used. The thumb and forefinger of the left hand were used to elevate a fold of skin and subcutaneous fat about 1 cm away from the measurement point. The right hand was used to open the calipers and place them over the skinfold perpendicular (at right angles) to the long axis (length) of the fold (Figure 1a). This was approximately halfway between the top of the fold and the body surface.

The reading was taken after the dial slowed to a stop. The caliper was not left in position for more than 4 seconds, to prevent compression of the skinfold. Once a reading was taken, the calipers were released first before releasing the fold held by the left hand. The process was repeated for a total of 3 measurements to the nearest 0.2mm, to use the median result. When taking a measurement, the operator’s head was positioned to avoid errors due to parallax. All measurements were recorded directly on the data collection form. At the end of the measurements, a cleansing wipe was offered to the mother to remove any white eyeliner. The calipers were also cleaned with an antibacterial surface wipe after use.

## Knee-heel length measurement

Two different types of knemometer were used. A custom-built electronic knemometer was used initially and compared to an adapted knemometer, which did not require batteries.

Custom-built electronic knemometer

The custom-build electronic knemometer was constructed with a digital caliper with a 0.01mm resolution (Mitutoyo, Neuss, Germany) mounted with knee and heel caps cast in hard plastic. The knemometer developed by Kim F Michaelsen has been used in other studies(2-4).

Knee-heel length was measured with the child staying in the ‘bear-hug’ position as used for the skinfold thicknesses. The left knee was bent at 90^0^ (figure 1c) with the leg free of clothing including socks. The lateral condyle of the knee was marked with a white eyebrow pencil. The lower arm of the knee-heel instrument was placed under the heel, using the Data Collector’s left hand. The lateral side of the foot was placed close to the “side” of the measuring arm (figure 1). The Data Collector’s right hand moved the upper measuring arm downwards until it touched the knee. After applying gentle pressure, a reading was performed digitally by pressing the record button to set the value. A series of 3 readings were performed and the median reading used for analysis. The instrument was removed between each measurement.

Adapted knemometer

The manual knemometer was made by reducing the lengths of both arms of a commercially available abdominal caliper (<https://holtain.co.uk/abdo.php>). It was important to ensure all fittings were tightly screwed together (particularly on the base) before using it. The positioning and measurement technique was identical to the electronic knemometer. The median of 3 readings was used, with the manual knemometer removed following each measurement.

Comparison of knemometer techniques

67 measurements were performed on children with both the custom-built and manual knemometers for comparison. The correlation between the custom-built and adapted knemometer was 0.9951. A Bland-Altman plot showed no significant bias, with a mean difference of -0.013 mm and 95% limits of agreement were ±2.4mm (Figure S2). Where possible, the manual knemometer data were used for analysis, because this was cheaper and easier to construct. However, electronic knemometer data were used when manual knemometer data were not available.

**S3 Fig:** Bland-Altman plot comparing custom-built and adapted knemometers, showing mean difference and 95% limits of agreement.

## Bioimpedance measurement

The Bodystat 1500 bioimpedance machine was carefully transported in a custom-made foam case to protect it whilst being transported on a data collector’s motorbike. At the start of each measurement, the machine was calibrated and the calibration value recorded. The foam mat was unfolded so that the child could get familiar with the mat and become used to lying on it. If the child did not like the mat, but could lie on a family cloth or other material, this was acceptable as long as it was not metal and not wet. If the child continued to be upset, occasionally the data collector waited until the child fell asleep. The child’s position was continually monitored, including ensuring that no other objects were touching the child.

The dorsal surface of the hand and foot were cleaned with wipes to ensure good contact between electrodes and skin. Due to the small size of the hands and feet in 2 year olds, electrodes were cut vertically in half. One electrode was placed just behind the knuckles and the other at least 3 cm away in at the wrist. A second pair of electrodes were placed on the dorsal surface of the right foot, behind the toes and at least 3cm away near the ankle. A 3cm card was provided to ensure the distance was far enough apart.

The child lay flat on the ground, with legs slightly apart and arms on the side of the body with palms facing down and away from the body (see Figure 1b). If necessary toys were used to distract the child to keep them lying down and the arms and legs straight. The test number and raw bioimpedance readings were recorded on a proforma for 2 measurements. Readings were then repeated if there was poor repeatability (duplicate value differences  >0.3^0^ for PA, and ≥6.0 Ω for either resistance (R) or reactance (Xc)) until agreement was reached. The lead wires and electrodes were then removed from the child.

## Derivation of model 4

Model 4 was derived as follows: Trial factors such as study nurse, calendar age of measurement, trial arm were all kept from model 3. Applying a limit of 10 observations per covariate (or divisions within the continuous variables) gave an available total of 22 parameters for adjustment, of which 20 parameters were used. These represented the following additional variables for model 4: Socioeconomic status, measured by wealth quintile, and nutritional insecurity, measured by the coping strategies index (CSI). Household size was also included as it has a complex relationship to socioeconomic status and nutritional insecurity. Maternal height was included due to its impact on genetic influence. Maternal education was also included as it represented an aspect of nurturing care. Maternal MUAC during pregnancy provided a measure of nutrition during pregnancy. The Edinburgh Postnatal Depression Scale (EPDS) was included as it reflects maternal mental health, which may influence child-rearing skills and nurturing. Maternal religion was included by categories of apostolic, Christian or other, as this had previously been shown to have an impact on child growth(5).

The following parameters were considered but not included in model 4: Birthweight and length at 1 month were not included because they were likely to be affected by HIV-exposure and hence were on the causal pathway (Table 1). Maternal age was not included as it related to maternal education and maternal socioeconomic status. Marital status was not included as this related mainly to socioeconomic status, coping strategies index and maternal education.

1.	Evans C, Chasekwa B, Ntozini R, Majo FD, Mutasa K, Tavengwa N, et al. Mortality, Human Immunodeficiency Virus (HIV) Transmission, and Growth in Children Exposed to HIV in Rural Zimbabwe. Clinical Infectious Diseases. 2021;72(4):586-94. doi: 10.1093/cid/ciaa076.

2.	Kamugisha JGK, Lanyero B, Nabukeera-Barungi N, Ritz C, Mølgaard C, Michaelsen KF, et al. Weight-for-Height Z-score Gain during Inpatient Treatment and Subsequent Linear Growth during Outpatient Treatment of Young Children with Severe Acute Malnutrition: A Prospective Study from Uganda. Current Developments in Nutrition. 2021;5(10):nzab118. doi: 10.1093/cdn/nzab118.

3.	Pesu H, Mutumba R, Mbabazi J, Olsen MF, Mølgaard C, Michaelsen KF, et al. The Role of Milk Protein and Whey Permeate in Lipid-based Nutrient Supplements on the Growth and Development of Stunted Children in Uganda: A Randomized Trial Protocol (MAGNUS). Current Developments in Nutrition. 2021;5(5):nzab067. doi: 10.1093/cdn/nzab067.

4.	Skau JK, Touch B, Chhoun C, Chea M, Unni US, Makurat J, et al. Effects of animal source food and micronutrient fortification in complementary food products on body composition, iron status, and linear growth: a randomized trial in Cambodia. The American journal of clinical nutrition. 2015;101(4):742-51. doi: 10.3945/ajcn.114.084889.

5.	Humphrey JH, Mbuya MNN, Ntozini R, Moulton LH, Stoltzfus RJ, Tavengwa NV, et al. Independent and combined effects of improved water, sanitation, and hygiene, and improved complementary feeding, on child stunting and anaemia in rural Zimbabwe: a cluster-randomised trial. The Lancet Global Health. 2019;7(1):e132-e47. doi: 10.1016/S2214-109X(18)30374-7.
