# Supplementary material for: Body composition of children born HIV-exposed uninfected in rural Zimbabwe
Source: PLoS One. 2026 Sep 11;21(9):e0319542. doi: 10.1371/journal.pone.0319542 (PMC13567772; doi:10.1371/journal.pone.0319542)
Supplement: Supplementary file 2 [file pone.0319542.s004.docx]

ONE-D-25-05396

Body composition of children born HIV-free in rural Zimbabwe

PLOS ONE

**Dear Dr. Fanuto,**

**Many thanks for to you and the reviewers for your constructive feedback. We have answered the comments below and enclose a revised cover letter, manuscript, title page, rebuttal letter, figure 2, copyright form, dataset and coding. We look forward to hearing from you.**

**Thanks again**

**Kindest regards**

**Dr Joe D Piper**

PLOS ONE

Journal Requirements:

When submitting your revision, we need you to address these additional requirements.

1. Please ensure that your manuscript meets PLOS ONE's style requirements, including those for file naming. The PLOS ONE style templates can be found at

<https://journals.plos.org/plosone/s/file?id=wjVg/PLOSOne_formatting_sample_main_body.pdf> and

[https://journals.plos.org/plosone/s/file?id=ba62/PLOSOne_formatting_sample_title_authors_affiliations.pdf](https://eur01.safelinks.protection.outlook.com/?url=https%3A%2F%2Ftrack.editorialmanager.com%2FCL0%2Fhttps%3A%252F%252Fjournals.plos.org%252Fplosone%252Fs%252Ffile%253Fid%3Dba62%252FPLOSOne_formatting_sample_title_authors_affiliations.pdf%2F1%2F010f0197aff5da3e-a979b144-5270-472b-a7f4-0a253c9a8c43-000000%2Fs4CwUVwRw2x_JjraeV8V-2iB8tZ7XHSoOEmzFW3reDE%3D217&data=05%7C02%7Cj.piper%40qmul.ac.uk%7Cb735d3b588d84251583208ddb53f6c2a%7C569df091b01340e386eebd9cb9e25814%7C0%7C0%7C638866006699385412%7CUnknown%7CTWFpbGZsb3d8eyJFbXB0eU1hcGkiOnRydWUsIlYiOiIwLjAuMDAwMCIsIlAiOiJXaW4zMiIsIkFOIjoiTWFpbCIsIldUIjoyfQ%3D%3D%7C0%7C%7C%7C&sdata=sr5fwagwZwKHQB72ZKvuUqqIaoxGbegtv7t7nch1LDQ%3D&reserved=0)

**The manuscript and title page have been updated.**

2. Please note that PLOS ONE has specific guidelines on code sharing for submissions in which author-generated code underpins the findings in the manuscript. In these cases, we expect all author-generated code to be made available without restrictions upon publication of the work. Please review our guidelines at <https://journals.plos.org/plosone/s/materials-and-software-sharing#loc-sharing-code> and ensure that your code is shared in a way that follows best practice and facilitates reproducibility and reuse.

**Thank you the stata code has been uploaded.**

3. We note that the grant information you provided in the ‘Funding Information’ and ‘Financial Disclosure’ sections do not match.

**Apologies this has been corrected.**

When you resubmit, please ensure that you provide the correct grant numbers for the awards you received for your study in the ‘Funding Information’ section.

4. Thank you for stating in your Funding Statement:
“Equipment for the body composition substudy was supported by Barts Charity, UK (https://www.bartscharity.org.uk/). JP was funded by an academic clinical fellowship provided by NIHR (ACF-2015-19-002) (https://www.nihr.ac.uk/) . The SHINE trial was funded by the Bill & Melinda Gates Foundation (https://www.gatesfoundation.org/) (OPP1021542 to Johns Hopkins Bloomberg School of Public Health and OPP1143707 to Zvitambo Institute for Maternal and Child Health Research), the UK Department for International Development (https://www.gov.uk/government/organisations/department-for-international-development), the Wellcome Trust https://wellcome.org/  (093768/Z/10/Z and 108065/Z/15/Z), the Swiss Agency for Development and Cooperation (https://www.eda.admin.ch/eda/en/fdfa/fdfa/organisation-fdfa/directorates-divisions/sdc.html) (8106727), UNICEF (https://www.unicef.org/) (PCA-2017-0002), and the US National Institutes of Health (https://www.nih.gov/) (R01 HD060338/HD/NICHD). The funders had no role in study design, data collection and analysis, decision to publish, or preparation of the manuscript.”

Please provide an amended statement that declares *all* the funding or sources of support (whether external or internal to your organization) received during this study, as detailed online in our guide for authors at http://journals.plos.org/plosone/s/submit-now. Please also include the statement “There was no additional external funding received for this study.” in your updated Funding Statement.

**This additional statement has been added to the cover letter and funding statement.**
Please include your amended Funding Statement within your cover letter. We will change the online submission form on your behalf.

5. In the online submission form, you indicated that [Data will be freely available as individual participant data on ClinEpiDB with an accompanying data dictionary at [http://ClinEpiDB.org](http://clinepidb.org) from 2026. Researchers must agree to the policies and comply with the mechanism of ClinepiDB to access data housed on this platform. Prior to that time, data are available upon reasonable request from the Zvitambo Institute for Maternal and Child Health Research, by contacting Dr Robert Ntozini ([r.ntozini@zvitambo.com](mailto:r.ntozini@zvitambo.com))].
All PLOS journals now require all data underlying the findings described in their manuscript to be freely available to other researchers, either 1. In a public repository, 2. Within the manuscript itself, or 3. Uploaded as supplementary information.
This policy applies to all data except where public deposition would breach compliance with the protocol approved by your research ethics board. If your data cannot be made publicly available for ethical or legal reasons (e.g., public availability would compromise patient privacy), please explain your reasons on resubmission and your exemption request will be escalated for approval. **Thank you: Anonymised data for this paper has now been uploaded as supplementary information.**

6. Please include your full ethics statement in the ‘Methods’ section of your manuscript file. In your statement, please include the full name of the IRB or ethics committee who approved or waived your study, as well as whether or not you obtained informed written or verbal consent. If consent was waived for your study, please include this information in your statement as well. **This has been included in methods**

7. We note that Figure 1 in your submission contain copyrighted images. All PLOS content is published under the Creative Commons Attribution License (CC BY 4.0), which means that the manuscript, images, and Supporting Information files will be freely available online, and any third party is permitted to access, download, copy, distribute, and use these materials in any way, even commercially, with proper attribution. For more information, see our copyright guidelines: <http://journals.plos.org/plosone/s/licenses-and-copyright>.

We require you to either (1) present written permission from the copyright holder to publish these figures specifically under the CC BY 4.0 license, or (2) remove the figures from your submission: **JDP is the copyright holder of these images and has provided the contact permission form.**

a. You may seek permission from the original copyright holder of Figure 1 to publish the content specifically under the CC BY 4.0 license.

We recommend that you contact the original copyright holder with the Content Permission Form (<http://journals.plos.org/plosone/s/file?id=7c09/content-permission-form.pdf>) and the following text:
“I request permission for the open-access journal PLOS ONE to publish XXX under the Creative Commons Attribution License (CCAL) CC BY 4.0 (<http://creativecommons.org/licenses/by/4.0/>). Please be aware that this license allows unrestricted use and distribution, even commercially, by third parties. Please reply and provide explicit written permission to publish XXX under a CC BY license and complete the attached form.”

Please upload the completed Content Permission Form or other proof of granted permissions as an "Other" file with your submission.

In the figure caption of the copyrighted figure, please include the following text: “Reprinted from [ref] under a CC BY license, with permission from [name of publisher], original copyright [original copyright year].” **This statement has been added to the caption for figure 1.**

b. If you are unable to obtain permission from the original copyright holder to publish these figures under the CC BY 4.0 license or if the copyright holder’s requirements are incompatible with the CC BY 4.0 license, please either i) remove the figure or ii) supply a replacement figure that complies with the CC BY 4.0 license. Please check copyright information on all replacement figures and update the figure caption with source information. If applicable, please specify in the figure caption text when a figure is similar but not identical to the original image and is therefore for illustrative purposes only.

**Reviewers' comments:**

Reviewer's Responses to Questions

**Comments to the Author**

5. Review Comments to the Author

Please use the space provided to explain your answers to the questions above. You may also include additional comments for the author, including concerns about dual publication, research ethics, or publication ethics. (Please upload your review as an attachment if it exceeds 20,000 characters)

**Reviewer #1**: Overall, this is an interesting paper showing compelling differences in body composition by HIV status; however, the paper can be improved. A better explanation of rationale for each of the analyses and potentially address multiple testing. Suggest revising the results and discussion to improve flow, and also tempering some of the findings, given the additional papers that were overlooked in this area in the published literature.

Abstract
- Add objective and potentially hypothesis.

**The objective is now defined in the abstract**
- Results – confusion about timing **in line 15**. Birthweight is at birth, but are all other variables reported at 2 years.

**Thank you for this, birthweight has been moved to a separate sentence on line 20 to make this clearer.**

Introduction
- There are several other studies that have reported on body composition in young infants in LMIC, including a paper by Lane that included repeated measures of anthropometry and skinfold thickness until 12-months, and another by Rickman et al that examined growth and body composition up to 23 months. **These additional papers have been included**
o Lane et al. HIV-Exposed, Uninfected Infants in Uganda Experience Poorer Growth and Body Composition Trajectories than HIV-Unexposed Infants. JAIDS Journal of Acquired Immune Deficiency Syndromes 85(2):p 138-147, October 1, 2020. | DOI: 10.1097/QAI.0000000000002428 <https://journals.lww.com/jaids/fulltext/2020/10010/HIV_Exposed,_Uninfected_Infants_in_Uganda.3.aspx?context=LatestArticles>
o Rickman et al. Body Composition Trajectories During the First 23 Months of Life Differ by HIV Exposure Among Infants in Western Kenya: A Prospective Study. J Nutr. 2023 Jan;153(1):331-339. doi: 10.1016/j.tjnut.2022.11.010. Epub 2022 Dec 21.<https://www.sciencedirect.com/science/article/pii/S0022316622131287>

**Thank you for this: the introduction has been updated with these references in lines 63 to 65 and Jumare et al. 2019, Rosala et al. 2017 and le Roux 2019 added in line 43 that measured poorer postatal growth**

- Why was age 2 selected, is there a particular reason? Are there any hypotheses? What do the different measures of body composition reflect?

**This has been clarified in that 2 years was part of a substudy measuring neurodevelopment in lines 80-81. The hypothesis has been included and stated that CBHF would have worse growth in both fat and lean mass, and the measures for this have been described in lines 83-85. 2 years (the endpoint of the first 1000 days) is a time of peak stunting, when much of the trajectory of future growth has been discussed. The first thousand days is a crucial window for generating the trajectory of both adipocyte number and adipose depots, as mentioned in the discussion (line 409).**
- I am confused about the interaction sentence, perhaps this can be clarified.

**Additional clarification has been given in lines 93.**

Methods-
- Children received WASH and/or IYCF intervention. Was this incorporated into analyses?

**This was incorporated into analyses in adjusted models as described in line 185 of the methods section.**

- Has this BIA device been validated in this or a similar population?

**This BIA device has been extensively validated in children [1] using a range of techniques [2]. These references have been added. Of note, the lean mass and impedance indices give relative measures of lean mass within a population so do not rely on calibrating equations for a specific population [3]. This has been clarified within the text and further links to this citation added on line 160 and 172.**

- Why were these skinfold sites selected?

**Triceps and subscapular are routine sites for the measurement of peripheral and central skinfold thickness, respectively. Central fat is linked more closely with pathogen load in ecogeographical analysis, whilst peripheral fat may be affected by HIV-exposure. Additionally, calf skinfolds were also measured to complement the knee-heel length and calf circumference measurements as linear growth restriction can particularly affect the limb fat distribution[4].**

- Are infants still being breastfed? Were there any protocols around hydration for the study measurements.

**We did not specifically ask whether the infants in this substudy were still breastfed and this has been added as a limitation; however, the majority of children in Zimbabwe have stopped being breastfed by this age. There were no specific protocols for the hydration measurements for these 2 year olds because it was more important they were calm for the measurement; this has been added to line 164.**

- What was the variability in age for the child measures at 2 years?

**The variability in age was small because children had to be enrolled to this substudy between 102-112 weeks of age. A standard deviation in months (SD 0.4) has been added on line 219 to provide the same units as table 2.**

- Statistics
o What was clustering by? By study site or something else?

**Clustering was by the original cluster randomized trial design, which randomized groups of 1-4 village health workers who covered a specific geographical area [5]. A line explaining this has been added at 221.**

o Why calendar month at birth? To account for seasonality? Add more details.

**Calendar month at birth was added to account for seasonality and this has been added to the paper (line 201).**

o The rationale for interaction terms and explanation of the analysis conducted are really unclear.

**This has been clarified in lines 208-213.**

Results/Discussion
- Generally, tables and text should be supportive of each other but not directly repeating findings; suggest revising results section for clarity and flow. Overall the organization of the results could be much improved. **We have removed findings that were repeated in tables 1 and 3 and kept some key comparisons from supplementary table S1, and the difference in birthweight, in the revised text to help readers in following the key findings.**

- There also seem to be results shown that are not described in the methods, or overall objective for the paper.. for example: “line 214: Total skinfolds were not significantly associated with unit increases in LAZ”. This analysis is not described clearly in the methods and the rationale for examining it could be much clearer. **This has been clarified in both the introduction (line 93) and methods (line 196).**

- Was there information on gestational duration? Could size for gestational age be calculated with an appropriate reference? Was size for gestational age different by HIV status in the sample?

**Gestational duration was not reliable within this cohort because no ultrasound dating was available in the district. Therefore, size for gestational age was not possible to be measured. This has been added within limitations in line 416-417.**

- Figure 2- suggest revising the colors to not **be blue and red**, perhaps to different shapes?

**Figure 2 has been redrawn to use shapes rather than colours to distinguish CBHF from CHU.**

- Table 3 is showing results from individual regression models for each result shown?

**Yes this is correct. This has been added in the footer for table 3.**

- From my understanding of these findings are not supported by the data shown.. Line 300: “We also found no evidence of interaction between HIV exposure and birthweight, suggesting that despite CBHF being lighter at birth, the observed differences in body composition at 2 years of age were not solely due to antenatal growth restriction.”

**This has been revised to explain the interaction between birthweight and HIV exposure when split for body composition outcomes in line 358.**

- Line 357: I believe the populations in Lane and Rickman’s studies also had a high burden of stunting

**This has been corrected to include these studies.**

- Is there information on breastfeeding and infant feeding in this population? Could these practices also influence the findings? **Exclusive breastfeeding within the cohort remained high due to an intensive breastfeeding intervention, as previously reported[6], with reported breastfeeding rates >96% in all arms at age 12 months[7], added in line 469.**

**Reviewer #2**: This is a well written manuscript. It is easy to read. The authors introduce well the subject, comprehensively describe the methods, present the results in a very clear fashion and the discussion is very good. A minor comment is that p-values should be added on table 2. Also, in addition to convenience sampling, the small number of CBHF should be mentioned as a limitation given the number of variables included in adjusted models. Finally, we suggest to include in the conclusion a list of research needed to enhance the understanding of the mechanisms leading to the observed low lean and fat masses and strengthen the evidence.

**We thank the Reviewer for these comments. P-values have been added to table 2 (which are also in table 3 for the unadjusted model). The small number of CBHF has been included as a limitation. Drawing on papers from the introduction, a paragraph has been added for mechanistic evidence to the discussion and summarised in the conclusion.**

PACE, please email PLOS at [figures@plos.org](mailto:figures@plos.org). Please note that Supporting Information files do not need this step.

**References**

1.	Nightingale CM, Rudnicka AR, Owen CG, Donin AS, Newton SL, Furness CA, et al. Are ethnic and gender specific equations needed to derive fat free mass from bioelectrical impedance in children of South asian, black african-Caribbean and white European origin? Results of the assessment of body composition in children study. PLoS ONE. 2013;8(10):e76426. Epub 20131018. doi: 10.1371/journal.pone.0076426. PubMed PMID: 24204625; PubMed Central PMCID: PMCPMC3799736.

2.	Parker L, Reilly JJ, Slater C, Wells JCK, Pitsiladis Y. Validity of Six Field and Laboratory Methods for Measurement of Body Composition in Boys. Obesity Research. 2003;11(7):852-8. doi: <https://doi.org/10.1038/oby.2003.117>.

3.	Wells JC, Williams JE, Fewtrell M, Singhal A, Lucas A, Cole TJ. A simplified approach to analysing bio-electrical impedance data in epidemiological surveys. Int J Obes (Lond). 2007;31(3):507-14. Epub 20060808. doi: 10.1038/sj.ijo.0803441. PubMed PMID: 16894361.

4.	Nicholson L, Chisenga M, Siame J, Kasonka L, Filteau S. Growth and health outcomes at school age in HIV-exposed, uninfected Zambian children: follow-up of two cohorts studied in infancy. BMC Pediatrics. 2015;15:66-. doi: 10.1186/s12887-015-0386-8. PubMed PMID: 26048411.

5.	Humphrey JH, Jones AD, Manges A, Mangwadu G, Maluccio JA, Mbuya MN, et al. The Sanitation Hygiene Infant Nutrition Efficacy (SHINE) Trial: Rationale, Design, and Methods. Clin Infect Dis. 2015;61 Suppl 7:S685-702. Epub 2015/11/26. doi: 10.1093/cid/civ844. PubMed PMID: 26602296; PubMed Central PMCID: PMCPMC4657589.

6.	Mbuya MNN, Matare CR, Tavengwa NV, Chasekwa B, Ntozini R, Majo FD, et al. Early Initiation and Exclusivity of Breastfeeding in Rural Zimbabwe: Impact of a Breastfeeding Intervention Delivered by Village Health Workers. Current Developments in Nutrition. 2019;3(4). doi: 10.1093/cdn/nzy092.

7.	Humphrey JH, Mbuya MNN, Ntozini R, Moulton LH, Stoltzfus RJ, Tavengwa NV, et al. Independent and combined effects of improved water, sanitation, and hygiene, and improved complementary feeding, on child stunting and anaemia in rural Zimbabwe: a cluster-randomised trial. The Lancet Global Health. 2019;7(1):e132-e47. doi: 10.1016/S2214-109X(18)30374-7.
