# Supplementary material for: Body composition of children born HIV-exposed uninfected in rural Zimbabwe
Source: PLoS One. 2026 Sep 11;21(9):e0319542. doi: 10.1371/journal.pone.0319542 (PMC13567772; doi:10.1371/journal.pone.0319542)
Supplement: Supplementary file 3 [file pone.0319542.s005.docx]

ONE-D-25-05396

Body composition of children born HIV-exposed uninfected in rural Zimbabwe

PLOS ONE

**Dear Dr. Fanuto,**

**Many thanks for to you and the reviewers for your constructive feedback. We have answered the comments below and enclose a revised cover letter, manuscript, title page, rebuttal letter, copyright form and figure 2. We have updated the title to reflect the more commonly used phrase “Children born HIV-exposed uninfected (CHEU).” We look forward to hearing from you.**

**Thanks again**

**Kindest regards**

**Dr Joe D Piper**

Please submit your revised manuscript by Aug 23 2026 11:59PM. If you will need more time than this to complete your revisions, please reply to this message or contact the journal office at [plosone@plos.org](mailto:plosone@plos.org). When you're ready to submit your revision, log on to <https://www.editorialmanager.com/pone/> and select the 'Submissions Needing Revision' folder to locate your manuscript file.

Please include the following items when submitting your revised manuscript:

- A letter that responds to each point raised by the academic editor and reviewer(s). You should upload this letter as a separate file labeled 'Response to Reviewers'.
- A marked-up copy of your manuscript that highlights changes made to the original version. You should upload this as a separate file labeled 'Revised Manuscript with Track Changes'.
- An unmarked version of your revised paper without tracked changes. You should upload this as a separate file labeled 'Manuscript'.

If you would like to make changes to your financial disclosure, please include your updated statement in your cover letter. Guidelines for resubmitting your figure files are available below the reviewer comments at the end of this letter.

If applicable, we recommend that you deposit your laboratory protocols in protocols.io to enhance the reproducibility of your results. Protocols.io assigns your protocol its own identifier (DOI) so that it can be cited independently in the future. For instructions see: [https://journals.plos.org/plosone/s/submission-guidelines#loc-laboratory-protocols](https://eur01.safelinks.protection.outlook.com/?url=https%3A%2F%2Ftrack.editorialmanager.com%2FCL0%2Fhttps%3A%252F%252Fjournals.plos.org%252Fplosone%252Fs%252Fsubmission-guidelines%2523loc-laboratory-protocols%2F1%2F010f019f49c4f3c7-228c46a7-a4a7-492b-ad87-f2ee976b1a5c-000000%2Fz3nClCpvTAbkpQS1qL9LfHsRZWBzeaqkuuDv1qaKpUE%3D258&data=05%7C02%7Cj.piper%40qmul.ac.uk%7Ce35f260565a6499eb0ee08dede27962f%7C569df091b01340e386eebd9cb9e25814%7C0%7C0%7C639192458796702141%7CUnknown%7CTWFpbGZsb3d8eyJFbXB0eU1hcGkiOnRydWUsIlYiOiIwLjAuMDAwMCIsIlAiOiJXaW4zMiIsIkFOIjoiTWFpbCIsIldUIjoyfQ%3D%3D%7C0%7C%7C%7C&sdata=9KbDEFG0HzIGtBQ7dnrahj1dtrL%2B%2Fd0sUvJwttakH0s%3D&reserved=0). Additionally, PLOS ONE offers an option for publishing peer-reviewed Lab Protocol articles, which describe protocols hosted on protocols.io. Read more information on sharing protocols at [https://plos.org/protocols?utm_medium=editorial-email&utm_source=authorletters&utm_campaign=protocols](https://eur01.safelinks.protection.outlook.com/?url=https%3A%2F%2Ftrack.editorialmanager.com%2FCL0%2Fhttps%3A%252F%252Fplos.org%252Fprotocols%253Futm_medium%3Deditorial-email%2526utm_source%3Dauthorletters%2526utm_campaign%3Dprotocols%2F1%2F010f019f49c4f3c7-228c46a7-a4a7-492b-ad87-f2ee976b1a5c-000000%2FCB_wpz33W-CaQlQbBTa5IB3fztvFU_HOTnKRZt0dWiw%3D258&data=05%7C02%7Cj.piper%40qmul.ac.uk%7Ce35f260565a6499eb0ee08dede27962f%7C569df091b01340e386eebd9cb9e25814%7C0%7C0%7C639192458796728173%7CUnknown%7CTWFpbGZsb3d8eyJFbXB0eU1hcGkiOnRydWUsIlYiOiIwLjAuMDAwMCIsIlAiOiJXaW4zMiIsIkFOIjoiTWFpbCIsIldUIjoyfQ%3D%3D%7C0%7C%7C%7C&sdata=cHsiuCHakP1JdE1KyhwYbQ3JsvicK8eY0Ccnf95aLUk%3D&reserved=0).

As the corresponding author, your ORCID iD is verified in the submission system and will appear in the published article. PLOS supports the use of ORCID, and we encourage all coauthors to register for an ORCID iD and use it as well. Please encourage your coauthors to verify their ORCID iD within the submission system before final acceptance, as unverified ORCID iDs will not appear in the published article. *Only* the individual author can complete the verification step; PLOS staff *cannot* verify ORCID iDs on behalf of authors.

We look forward to receiving your revised manuscript.

Kind regards,

Emma K. Kalk
Academic Editor
PLOS One

Journal Requirements:

If the reviewer comments include a recommendation to cite specific previously published works, please review and evaluate these publications to determine whether they are relevant and should be cited. There is no requirement to cite these works unless the editor has indicated otherwise. 

Please review your reference list to ensure that it is complete and correct. If you have cited papers that have been retracted, please include the rationale for doing so in the manuscript text, or remove these references and replace them with relevant current references. Any changes to the reference list should be mentioned in the rebuttal letter that accompanies your revised manuscript. If you need to cite a retracted article, indicate the article’s retracted status in the References list and also include a citation and full reference for the retraction notice.


[Note: HTML markup is below. Please do not edit.]

Reviewers' comments:

Reviewer's Responses to Questions

**Comments to the Author**

1. If the authors have adequately addressed your comments raised in a previous round of review and you feel that this manuscript is now acceptable for publication, you may indicate that here to bypass the “Comments to the Author” section, enter your conflict of interest statement in the “Confidential to Editor” section, and submit your "Accept" recommendation.

Reviewer #1: All comments have been addressed

Reviewer #2: All comments have been addressed

Reviewer #3: (No Response)

2. Is the manuscript technically sound, and do the data support the conclusions?

The manuscript must describe a technically sound piece of scientific research with data that supports the conclusions. Experiments must have been conducted rigorously, with appropriate controls, replication, and sample sizes. The conclusions must be drawn appropriately based on the data presented.

Reviewer #1: Yes

Reviewer #2: Yes

Reviewer #3: Yes

3. Has the statistical analysis been performed appropriately and rigorously?

Reviewer #1: Yes

Reviewer #2: Yes

Reviewer #3: Yes

4. Have the authors made all data underlying the findings in their manuscript fully available?

The [PLOS Data policy](https://eur01.safelinks.protection.outlook.com/?url=http%3A%2F%2Ftrack.editorialmanager.com%2FCL0%2Fhttp%3A%252F%252Fwww.plosone.org%252Fstatic%252Fpolicies.action%2523sharing%2F1%2F010f019f49c4f3c7-228c46a7-a4a7-492b-ad87-f2ee976b1a5c-000000%2FluzSpagWUpyfHz9w52RWsEQbxgjLNYfj5-zrdOpPSBw%3D258&data=05%7C02%7Cj.piper%40qmul.ac.uk%7Ce35f260565a6499eb0ee08dede27962f%7C569df091b01340e386eebd9cb9e25814%7C0%7C0%7C639192458796739514%7CUnknown%7CTWFpbGZsb3d8eyJFbXB0eU1hcGkiOnRydWUsIlYiOiIwLjAuMDAwMCIsIlAiOiJXaW4zMiIsIkFOIjoiTWFpbCIsIldUIjoyfQ%3D%3D%7C0%7C%7C%7C&sdata=usDbfdrf6nN9NaHCMPFp8Bd%2FifdOLUrNWJJHt3zTV40%3D&reserved=0) requires authors to make all data underlying the findings described in their manuscript fully available without restriction, with rare exception (please refer to the Data Availability Statement in the manuscript PDF file). The data should be provided as part of the manuscript or its supporting information, or deposited to a public repository. For example, in addition to summary statistics, the data points behind means, medians and variance measures should be available. If there are restrictions on publicly sharing data—e.g. participant privacy or use of data from a third party—those must be specified.

Reviewer #1: Yes

Reviewer #2: Yes

Reviewer #3: Yes

5. Is the manuscript presented in an intelligible fashion and written in standard English?

PLOS ONE does not copyedit accepted manuscripts, so the language in submitted articles must be clear, correct, and unambiguous. Any typographical or grammatical errors should be corrected at revision, so please note any specific errors here.

Reviewer #1: Yes

Reviewer #2: Yes

Reviewer #3: Yes

6. Review Comments to the Author

Please use the space provided to explain your answers to the questions above. You may also include additional comments for the author, including concerns about dual publication, research ethics, or publication ethics. (Please upload your review as an attachment if it exceeds 20,000 characters)

Reviewer #1: The authors have sufficiently addressed my feedback, and the paper is now strengthened. It is an important contribution to the literature.

Reviewer #2: I am fully satisfied with the authors responses and the changes made to address my comments. I don't have additional comments.

Reviewer #3: Well and comprehensive written document with technically sound scientific data that supports the conclusions. Findings can be valuable for future public health research focusing on children born to HIV exposed mothers. Just oneconfirmation - was the equipment use for Bio-impedance analysis (Bodystat 1500 MDD Instrument) calibrated/needed to be calibrated after been transported by motorbike?

**For each visit, the Bodystat 1500 was calibrated after being transported by motorbike to the household (using the calibration resistor) and before being tested on the participants. This has been clarified as follows:**

**“**Once set up at the household, calibration was performed before each set of BIA measurements using the 500 Ohm calibration resistor provided (the acceptable range was 497 to 503 Ohms).”
