# Supplementary material for: Body composition of children born HIV-exposed uninfected in rural Zimbabwe
Source: PLoS One. 2026 Sep 11;21(9):e0319542. doi: 10.1371/journal.pone.0319542 (PMC13567772; doi:10.1371/journal.pone.0319542)
Supplement: Peer review history

# Peer review: Decision Letter 0

**DOI:** 10.1371/journal.pone.0319542.r001

**Authors:** Kazuo Funato

27 Jun 2025

Dear Dr. Piper,

Thank you for submitting your manuscript to PLOS ONE. After careful consideration, we feel that it has merit but does not fully meet PLOS ONE’s publication criteria as it currently stands. Therefore, we invite you to submit a revised version of the manuscript that addresses the points raised during the review process.

Please submit your revised manuscript by Aug 11 2025 11:59PM. If you will need more time than this to complete your revisions, please reply to this message or contact the journal office at plosone@plos.org. When you're ready to submit your revision, log on to https://www.editorialmanager.com/pone/ and select the 'Submissions Needing Revision' folder to locate your manuscript file.

- A rebuttal letter that responds to each point raised by the academic editor and reviewer(s). You should upload this letter as a separate file labeled 'Response to Reviewers'.
- A marked-up copy of your manuscript that highlights changes made to the original version. You should upload this as a separate file labeled 'Revised Manuscript with Track Changes'.
- An unmarked version of your revised paper without tracked changes. You should upload this as a separate file labeled 'Manuscript'.

If you would like to make changes to your financial disclosure, please include your updated statement in your cover letter. Guidelines for resubmitting your figure files are available below the reviewer comments at the end of this letter.

If applicable, we recommend that you deposit your laboratory protocols in protocols.io to enhance the reproducibility of your results. Protocols.io assigns your protocol its own identifier (DOI) so that it can be cited independently in the future. For instructions see: https://journals.plos.org/plosone/s/submission-guidelines#loc-laboratory-protocols. Additionally, PLOS ONE offers an option for publishing peer-reviewed Lab Protocol articles, which describe protocols hosted on protocols.io. Read more information on sharing protocols at https://plos.org/protocols?utm_medium=editorial-email&utm_source=authorletters&utm_campaign=protocols.

We look forward to receiving your revised manuscript.

Kind regards,

Kazuo Funato

Academic Editor

PLOS ONE

Journal Requirements:

When submitting your revision, we need you to address these additional requirements.

1. Please ensure that your manuscript meets PLOS ONE's style requirements, including those for file naming. The PLOS ONE style templates can be found at

https://journals.plos.org/plosone/s/file?id=wjVg/PLOSOne_formatting_sample_main_body.pdf and

https://journals.plos.org/plosone/s/file?id=ba62/PLOSOne_formatting_sample_title_authors_affiliations.pdf

2. Please note that PLOS ONE has specific guidelines on code sharing for submissions in which author-generated code underpins the findings in the manuscript. In these cases, we expect all author-generated code to be made available without restrictions upon publication of the work. Please review our guidelines at https://journals.plos.org/plosone/s/materials-and-software-sharing#loc-sharing-code and ensure that your code is shared in a way that follows best practice and facilitates reproducibility and reuse.

3. We note that the grant information you provided in the ‘Funding Information’ and ‘Financial Disclosure’ sections do not match.

When you resubmit, please ensure that you provide the correct grant numbers for the awards you received for your study in the ‘Funding Information’ section.

4. Thank you for stating in your Funding Statement:

“Equipment for the body composition substudy was supported by Barts Charity, UK (https://www.bartscharity.org.uk/). JP was funded by an academic clinical fellowship provided by NIHR (ACF-2015-19-002) (https://www.nihr.ac.uk/) . The SHINE trial was funded by the Bill & Melinda Gates Foundation (https://www.gatesfoundation.org/) (OPP1021542 to Johns Hopkins Bloomberg School of Public Health and OPP1143707 to Zvitambo Institute for Maternal and Child Health Research), the UK Department for International Development (https://www.gov.uk/government/organisations/department-for-international-development), the Wellcome Trust https://wellcome.org/  (093768/Z/10/Z and 108065/Z/15/Z), the Swiss Agency for Development and Cooperation (https://www.eda.admin.ch/eda/en/fdfa/fdfa/organisation-fdfa/directorates-divisions/sdc.html) (8106727), UNICEF (https://www.unicef.org/) (PCA-2017-0002), and the US National Institutes of Health (https://www.nih.gov/) (R01 HD060338/HD/NICHD). The funders had no role in study design, data collection and analysis, decision to publish, or preparation of the manuscript.”

Please provide an amended statement that declares *all* the funding or sources of support (whether external or internal to your organization) received during this study, as detailed online in our guide for authors at http://journals.plos.org/plosone/s/submit-now. Please also include the statement “There was no additional external funding received for this study.” in your updated Funding Statement.

Please include your amended Funding Statement within your cover letter. We will change the online submission form on your behalf.

5. In the online submission form, you indicated that [Data will be freely available as individual participant data on ClinEpiDB with an accompanying data dictionary at http://ClinEpiDB.org from 2026. Researchers must agree to the policies and comply with the mechanism of ClinepiDB to access data housed on this platform. Prior to that time, data are available upon reasonable request from the Zvitambo Institute for Maternal and Child Health Research, by contacting Dr Robert Ntozini (r.ntozini@zvitambo.com)].

All PLOS journals now require all data underlying the findings described in their manuscript to be freely available to other researchers, either 1. In a public repository, 2. Within the manuscript itself, or 3. Uploaded as supplementary information.

This policy applies to all data except where public deposition would breach compliance with the protocol approved by your research ethics board. If your data cannot be made publicly available for ethical or legal reasons (e.g., public availability would compromise patient privacy), please explain your reasons on resubmission and your exemption request will be escalated for approval.

6. Please include your full ethics statement in the ‘Methods’ section of your manuscript file. In your statement, please include the full name of the IRB or ethics committee who approved or waived your study, as well as whether or not you obtained informed written or verbal consent. If consent was waived for your study, please include this information in your statement as well.

7. We note that Figure 1 in your submission contain copyrighted images. All PLOS content is published under the Creative Commons Attribution License (CC BY 4.0), which means that the manuscript, images, and Supporting Information files will be freely available online, and any third party is permitted to access, download, copy, distribute, and use these materials in any way, even commercially, with proper attribution. For more information, see our copyright guidelines: http://journals.plos.org/plosone/s/licenses-and-copyright.

We require you to either (1) present written permission from the copyright holder to publish these figures specifically under the CC BY 4.0 license, or (2) remove the figures from your submission:

a. You may seek permission from the original copyright holder of Figure 1 to publish the content specifically under the CC BY 4.0 license.

We recommend that you contact the original copyright holder with the Content Permission Form (http://journals.plos.org/plosone/s/file?id=7c09/content-permission-form.pdf) and the following text:

“I request permission for the open-access journal PLOS ONE to publish XXX under the Creative Commons Attribution License (CCAL) CC BY 4.0 (http://creativecommons.org/licenses/by/4.0/). Please be aware that this license allows unrestricted use and distribution, even commercially, by third parties. Please reply and provide explicit written permission to publish XXX under a CC BY license and complete the attached form.”

Please upload the completed Content Permission Form or other proof of granted permissions as an "Other" file with your submission.

In the figure caption of the copyrighted figure, please include the following text: “Reprinted from [ref] under a CC BY license, with permission from [name of publisher], original copyright [original copyright year].”

b. If you are unable to obtain permission from the original copyright holder to publish these figures under the CC BY 4.0 license or if the copyright holder’s requirements are incompatible with the CC BY 4.0 license, please either i) remove the figure or ii) supply a replacement figure that complies with the CC BY 4.0 license. Please check copyright information on all replacement figures and update the figure caption with source information. If applicable, please specify in the figure caption text when a figure is similar but not identical to the original image and is therefore for illustrative purposes only.

Additional Editor Comments:

Dear Joe D Piper

Sorry for taking longer than expected. I had a difficulty in finding agreed reviewers for your manuscript. I finally received comments from two reviewers.

Outstanding two reviewers assessed your manuscript as positive concerns as well as major and minor changes. You are recommended to revise and resubmit according to reviewer’s comments.

We are looking forward to taking your revised manuscript.

Thank you for your consistent concern on PLOS ONE.

Kind regards,

Kazuo Funato, Ph.D. Academic Editor PLOS ONE

[Note: HTML markup is below. Please do not edit.]

Reviewers' comments:

Reviewer's Responses to Questions

**Comments to the Author**

1. Is the manuscript technically sound, and do the data support the conclusions?

Reviewer #1: Partly

Reviewer #2: Yes

**********

2. Has the statistical analysis been performed appropriately and rigorously?

Reviewer #1: No

Reviewer #2: Yes

**********

3. Have the authors made all data underlying the findings in their manuscript fully available?

The PLOS Data policy

Reviewer #1: Yes

Reviewer #2: Yes

**********

4. Is the manuscript presented in an intelligible fashion and written in standard English?

Reviewer #1: No

Reviewer #2: Yes

**********

Reviewer #1: Overall, this is an interesting paper showing compelling differences in body composition by HIV status; however, the paper can be improved. A better explanation of rationale for each of the analyses and potentially address multiple testing. Suggest revising the results and discussion to improve flow, and also tempering some of the findings, given the additional papers that were overlooked in this area in the published literature.

Abstract

- Add objective and potentially hypothesis

- Results – confusion about timing in line 15. Birthweight is at birth, but are all other variables reported at 2 years.

Introduction

- There are several other studies that have reported on body composition in young infants in LMIC, including a paper by Lane that included repeated measures of anthropometry and skinfold thickness until 12-months, and another by Rickman et al that examined growth and body composition up to 23 months.

o Lane et al. HIV-Exposed, Uninfected Infants in Uganda Experience Poorer Growth and Body Composition Trajectories than HIV-Unexposed Infants. JAIDS Journal of Acquired Immune Deficiency Syndromes 85(2):p 138-147, October 1, 2020. | DOI: 10.1097/QAI.0000000000002428 https://journals.lww.com/jaids/fulltext/2020/10010/HIV_Exposed,_Uninfected_Infants_in_Uganda.3.aspx?context=LatestArticles

o Rickman et al. Body Composition Trajectories During the First 23 Months of Life Differ by HIV Exposure Among Infants in Western Kenya: A Prospective Study. J Nutr. 2023 Jan;153(1):331-339. doi: 10.1016/j.tjnut.2022.11.010. Epub 2022 Dec 21. https://www.sciencedirect.com/science/article/pii/S0022316622131287

- Why was age 2 selected, is there a particular reason? Are there any hypotheses? What do the different measures of body composition reflect?

- I am confused about the interaction sentence, perhaps this can be clarified.

Methods-

- Children received WASH and/or IYCF intervention. Was this incorporated into analyses?

- Has this BIA device been validated in this or a similar population?

- Why were these skinfold sites selected?

- Are infants still being breastfed? Were there any protocols around hydration for the study measurements..

- What was the variability in age for the child measures at 2 years?

- Statistics

o What was clustering by? By study site or something else?

o Why calendar month at birth? To account for seasonality? Add more details.

o The rationale for interaction terms and explanation of the analysis conducted are really unclear.

Results/Discussion

- Generally, tables and text should be supportive of each other but not directly repeating findings; suggest revising results section for clarity and flow. Overall the organization of the results could be much improved.

- There also seem to be results shown that are not described in the methods, or overall objective for the paper.. for example: “line 214: Total skinfolds were not significantly associated with unit increases in LAZ”.. This analysis is not described clearly in the methods and the rationale for examining it could be much clearer

- Was there information on gestational duration? Could size for gestational age be calculated with an appropriate reference? Was size for gestational age different by HIV status in the sample?

- Figure 2- suggest revising the colors to not be blue and red, perhaps to different shapes?

- Table 3 is showing results from individual regression models for each result shown?

- From my understanding of these findings are not supported by the data shown.. Line 300: “We also found no evidence of interaction between HIV exposure and birthweight, suggesting that despite CBHF being lighter at birth, the observed differences in body composition at 2 years of age were not solely due to antenatal growth restriction.”

- Line 357: I believe the populations in Lane and Rickman’s studies also had a high burden of stunting

- Is there information on breastfeeding and infant feeding in this population? Could these practices also influence the findings?

Reviewer #2: This is a well written manuscript. It is easy to read. The authors introduce well the subject, comprehensively describe the methods, present the results in a very clear fashion and the discussion is very good. A minor comment is that p-values should be added on table 2. Also, in addition to convenience sampling, the small number of CBHF bshould be mentionned as a limitation given the number of variables included in adjusted models. Finally, we suggest to include in the conclusion a list of research needed to enhance the understanding of the mechanisms leading to the observed low lean and fat masses and strengthen the evidence.

**********

what does this mean?). If published, this will include your full peer review and any attached files.

If you choose “no”, your identity will remain anonymous but your review may still be made public.

**Do you want your identity to be public for this peer review?**  For information about this choice, including consent withdrawal, please see our Privacy Policy

Reviewer #1: No

Reviewer #2: **Yes:** Paluku Bahwere, PhD

**********

[NOTE: If reviewer comments were submitted as an attachment file, they will be attached to this email and accessible via the submission site. Please log into your account, locate the manuscript record, and check for the action link "View Attachments". If this link does not appear, there are no attachment files.]

While revising your submission, please upload your figure files to the Preflight Analysis and Conversion Engine (PACE) digital diagnostic tool, https://pacev2.apexcovantage.com/. PACE helps ensure that figures meet PLOS requirements. To use PACE, you must first register as a user. Registration is free. Then, login and navigate to the UPLOAD tab, where you will find detailed instructions on how to use the tool. If you encounter any issues or have any questions when using PACE, please email PLOS at figures@plos.org. Please note that Supporting Information files do not need this step.

# Author comment: Author response to Decision Letter 1

**DOI:** 10.1371/journal.pone.0319542.r002

29 Oct 2025

When submitting your revision, we need you to address these additional requirements.

1. Please ensure that your manuscript meets PLOS ONE's style requirements, including those for file naming. The PLOS ONE style templates can be found at

https://journals.plos.org/plosone/s/file?id=wjVg/PLOSOne_formatting_sample_main_body.pdf and

https://journals.plos.org/plosone/s/file?id=ba62/PLOSOne_formatting_sample_title_authors_affiliations.pdf

The manuscript and title page have been updated.

2. Please note that PLOS ONE has specific guidelines on code sharing for submissions in which author-generated code underpins the findings in the manuscript. In these cases, we expect all author-generated code to be made available without restrictions upon publication of the work. Please review our guidelines at https://journals.plos.org/plosone/s/materials-and-software-sharing#loc-sharing-code and ensure that your code is shared in a way that follows best practice and facilitates reproducibility and reuse.

Thank you the stata code has been uploaded.

3. We note that the grant information you provided in the ‘Funding Information’ and ‘Financial Disclosure’ sections do not match.

Apologies this has been corrected.

When you resubmit, please ensure that you provide the correct grant numbers for the awards you received for your study in the ‘Funding Information’ section.

4. Thank you for stating in your Funding Statement:

“Equipment for the body composition substudy was supported by Barts Charity, UK (https://www.bartscharity.org.uk/). JP was funded by an academic clinical fellowship provided by NIHR (ACF-2015-19-002) (https://www.nihr.ac.uk/) . The SHINE trial was funded by the Bill & Melinda Gates Foundation (https://www.gatesfoundation.org/) (OPP1021542 to Johns Hopkins Bloomberg School of Public Health and OPP1143707 to Zvitambo Institute for Maternal and Child Health Research), the UK Department for International Development (https://www.gov.uk/government/organisations/department-for-international-development), the Wellcome Trust https://wellcome.org/ (093768/Z/10/Z and 108065/Z/15/Z), the Swiss Agency for Development and Cooperation (https://www.eda.admin.ch/eda/en/fdfa/fdfa/organisation-fdfa/directorates-divisions/sdc.html) (8106727), UNICEF (https://www.unicef.org/) (PCA-2017-0002), and the US National Institutes of Health (https://www.nih.gov/) (R01 HD060338/HD/NICHD). The funders had no role in study design, data collection and analysis, decision to publish, or preparation of the manuscript.”

Please provide an amended statement that declares *all* the funding or sources of support (whether external or internal to your organization) received during this study, as detailed online in our guide for authors at http://journals.plos.org/plosone/s/submit-now. Please also include the statement “There was no additional external funding received for this study.” in your updated Funding Statement.

This additional statement has been added to the cover letter and funding statement.

Please include your amended Funding Statement within your cover letter. We will change the online submission form on your behalf.

5. In the online submission form, you indicated that [Data will be freely available as individual participant data on ClinEpiDB with an accompanying data dictionary at http://ClinEpiDB.org from 2026. Researchers must agree to the policies and comply with the mechanism of ClinepiDB to access data housed on this platform. Prior to that time, data are available upon reasonable request from the Zvitambo Institute for Maternal and Child Health Research, by contacting Dr Robert Ntozini (r.ntozini@zvitambo.com)].

All PLOS journals now require all data underlying the findings described in their manuscript to be freely available to other researchers, either 1. In a public repository, 2. Within the manuscript itself, or 3. Uploaded as supplementary information.

This policy applies to all data except where public deposition would breach compliance with the protocol approved by your research ethics board. If your data cannot be made publicly available for ethical or legal reasons (e.g., public availability would compromise patient privacy), please explain your reasons on resubmission and your exemption request will be escalated for approval. Thank you: Anonymised data for this paper has now been uploaded as supplementary information.

6. Please include your full ethics statement in the ‘Methods’ section of your manuscript file. In your statement, please include the full name of the IRB or ethics committee who approved or waived your study, as well as whether or not you obtained informed written or verbal consent. If consent was waived for your study, please include this information in your statement as well. This has been included in methods

7. We note that Figure 1 in your submission contain copyrighted images. All PLOS content is published under the Creative Commons Attribution License (CC BY 4.0), which means that the manuscript, images, and Supporting Information files will be freely available online, and any third party is permitted to access, download, copy, distribute, and use these materials in any way, even commercially, with proper attribution. For more information, see our copyright guidelines: http://journals.plos.org/plosone/s/licenses-and-copyright.

We require you to either (1) present written permission from the copyright holder to publish these figures specifically under the CC BY 4.0 license, or (2) remove the figures from your submission: JDP is the copyright holder of these images and has provided the contact permission form.

a. You may seek permission from the original copyright holder of Figure 1 to publish the content specifically under the CC BY 4.0 license.

We recommend that you contact the original copyright holder with the Content Permission Form (http://journals.plos.org/plosone/s/file?id=7c09/content-permission-form.pdf) and the following text:

“I request permission for the open-access journal PLOS ONE to publish XXX under the Creative Commons Attribution License (CCAL) CC BY 4.0 (http://creativecommons.org/licenses/by/4.0/). Please be aware that this license allows unrestricted use and distribution, even commercially, by third parties. Please reply and provide explicit written permission to publish XXX under a CC BY license and complete the attached form.”

Please upload the completed Content Permission Form or other proof of granted permissions as an "Other" file with your submission.

In the figure caption of the copyrighted figure, please include the following text: “Reprinted from [ref] under a CC BY license, with permission from [name of publisher], original copyright [original copyright year].” This statement has been added to the caption for figure 1.

b. If you are unable to obtain permission from the original copyright holder to publish these figures under the CC BY 4.0 license or if the copyright holder’s requirements are incompatible with the CC BY 4.0 license, please either i) remove the figure or ii) supply a replacement figure that complies with the CC BY 4.0 license. Please check copyright information on all replacement figures and update the figure caption with source information. If applicable, please specify in the figure caption text when a figure is similar but not identical to the original image and is therefore for illustrative purposes only.

Reviewers' comments:

Reviewer's Responses to Questions

Comments to the Author

5. Review Comments to the Author

Please use the space provided to explain your answers to the questions above. You may also include additional comments for the author, including concerns about dual publication, research ethics, or publication ethics. (Please upload your review as an attachment if it exceeds 20,000 characters)

Reviewer #1: Overall, this is an interesting paper showing compelling differences in body composition by HIV status; however, the paper can be improved. A better explanation of rationale for each of the analyses and potentially address multiple testing. Suggest revising the results and discussion to improve flow, and also tempering some of the findings, given the additional papers that were overlooked in this area in the published literature.

Abstract

- Add objective and potentially hypothesis.

The objective is now defined in the abstract

- Results – confusion about timing in line 15. Birthweight is at birth, but are all other variables reported at 2 years.

Thank you for this, birthweight has been moved to a separate sentence on line 20 to make this clearer.

Introduction

- There are several other studies that have reported on body composition in young infants in LMIC, including a paper by Lane that included repeated measures of anthropometry and skinfold thickness until 12-months, and another by Rickman et al that examined growth and body composition up to 23 months. These additional papers have been included

o Lane et al. HIV-Exposed, Uninfected Infants in Uganda Experience Poorer Growth and Body Composition Trajectories than HIV-Unexposed Infants. JAIDS Journal of Acquired Immune Deficiency Syndromes 85(2):p 138-147, October 1, 2020. | DOI: 10.1097/QAI.0000000000002428 https://journals.lww.com/jaids/fulltext/2020/10010/HIV_Exposed,_Uninfected_Infants_in_Uganda.3.aspx?context=LatestArticles

o Rickman et al. Body Composition Trajectories During the First 23 Months of Life Differ by HIV Exposure Among Infants in Western Kenya: A Prospective Study. J Nutr. 2023 Jan;153(1):331-339. doi: 10.1016/j.tjnut.2022.11.010. Epub 2022 Dec 21. https://www.sciencedirect.com/science/article/pii/S0022316622131287

Thank you for this: the introduction has been updated with these references in lines 63 to 65 and Jumare et al. 2019, Rosala et al. 2017 and le Roux 2019 added in line 43 that measured poorer postatal growth

- Why was age 2 selected, is there a particular reason? Are there any hypotheses? What do the different measures of body composition reflect?

This has been clarified in that 2 years was part of a substudy measuring neurodevelopment in lines 80-81. The hypothesis has been included and stated that CBHF would have worse growth in both fat and lean mass, and the measures for this have been described in lines 83-85. 2 years (the endpoint of the first 1000 days) is a time of peak stunting, when much of the trajectory of future growth has been discussed. The first thousand days is a crucial window for generating the trajectory of both adipocyte number and adipose depots, as mentioned in the discussion (line 409).

- I am confused about the interaction sentence, perhaps this can be clarified.

Additional clarification has been given in lines 93.

Methods-

- Children received WASH and/or IYCF intervention. Was this incorporated into analyses?

This was incorporated into analyses in adjusted models as described in line 185 of the methods section.

- Has this BIA device been validated in this or a similar population?

This BIA device has been extensively validated in children [1] using a range of techniques [2]. These references have been added. Of note, the lean mass and impedance indices give relative measures of lean mass within a population so do not rely on calibrating equations for a specific population [3]. This has been clarified within the text and further links to this citation added on line 160 and 172.

- Why were these skinfold sites selected?

Triceps and subscapular are routine sites for the measurement of peripheral and central skinfold thickness, respectively. Central fat is linked more closely with pathogen load in ecogeographical analysis, whilst peripheral fat may be affected by HIV-exposure. Additionally, calf skinfolds were also measured to complement the knee-heel length and calf circumference measurements as linear growth restriction can particularly affect the limb fat distribution[4].

- Are infants still being breastfed? Were there any protocols around hydration for the study measurements.

We did not specifically ask whether the infants in this substudy were still breastfed and this has been added as a limitation; however, the majority of children in Zimbabwe have stopped being breastfed by this age. There were no specific protocols for the hydration measurements for these 2 year olds because it was more important they were calm for the measurement; this has been added to line 164.

- What was the variability in age for the child measures at 2 years?

The variability in age was small because children had to be enrolled to this substudy between 102-112 weeks of age. A standard deviation in months (SD 0.4) has been added on line 219 to provide the same units as table 2.

- Statistics

o What was clustering by? By study site or something else?

Clustering was by the original cluster randomized trial design, which randomized groups of 1-4 village health workers who covered a specific geographical area [5]. A line explaining this has been added at 221.

o Why calendar month at birth? To account for seasonality? Add more details.

Calendar month at birth was added to account for seasonality and this has been added to the paper (line 201).

o The rationale for interaction terms and explanation of the analysis conducted are really unclear.

This has been clarified in lines 208-213.

Results/Discussion

- Generally, tables and text should be supportive of each other but not directly repeating findings; suggest revising results section for clarity and flow. Overall the organization of the results could be much improved. We have removed findings that were repeated in tables 1 and 3 and kept some key comparisons from supplementary table S1, and the difference in birthweight, in the revised text to help readers in following the key findings.

- There also seem to be results shown that are not described in the methods, or overall objective for the paper.. for example: “line 214: Total skinfolds were not significantly associated with unit increases in LAZ”. This analysis is not described clearly in the methods and the rationale for examining it could be much clearer. This has been clarified in both the introduction (line 93) and methods (line 196).

- Was there information on gestational duration? Could size for gestational age be calculated with an appropriate reference? Was size for gestational age different by HIV status in the sample?

Gestational duration was not reliable within this cohort because no ultrasound dating was available in the district. Therefore, size for gestational age was not possible to be measured. This has been added within limitations in line 416-417.

- Figure 2- suggest revising the colors to not be blue and red, perhaps to different shapes?

Figure 2 has been redrawn to use shapes rather than colours to distinguish CBHF from CHU.

- Table 3 is showing results from individual regression models for each result shown?

Yes this is correct. This has been added in the footer for table 3.

- From my understanding of these findings are not supported by the data shown.. Line 300: “We also found no evidence of interaction between HIV exposure and birthweight, suggesting that despite CBHF being lighter at birth, the observed differences in body composition at 2 years of age were not solely due to antenatal growth restriction.”

This has been revised to explain the interaction between birthweight and HIV exposure when split for body composition outcomes in line 358.

- Line 357: I believe the populations in Lane and Rickman’s studies also had a high burden of stunting

This has been corrected to include these studies.

- Is there information on breastfeeding and infant feeding in this population? Could these practices also influence the findings? Exclusive breastfeeding within the cohort remained high due to an intensive breastfeeding intervention, as previously reported[6], with reported breastfeeding rates >96% in all arms at age 12 months[7], added in line 469.

Reviewer #2: This is a well written manuscript. It is easy to read. The authors introduce well the subject, comprehensively describe the methods, present the results in a very clear fashion and the discussion is very good. A minor comment is that p-values should be added on table 2. Also, in addition

Attachment Submitted filename: 2_CBHF_editor_reviewer_reply.docx

# Peer review: Decision Letter 1

**DOI:** 10.1371/journal.pone.0319542.r003

**Authors:** Emma Kalk

9 Jul 2026

Dear Dr. Piper,

Thank you for submitting your manuscript to PLOS ONE. After careful consideration, we feel that it has merit but does not fully meet PLOS ONE’s publication criteria as it currently stands. Therefore, we invite you to submit a revised version of the manuscript that addresses the points raised during the review process.

-->Apologies for the delay. One small additional question only.

Was the equipment use for Bio-impedance analysis (Bodystat 1500 MDD Instrument) calibrated/needed to be calibrated after been transported by motorbike?

==============================?>

Please submit your revised manuscript by Aug 23 2026 11:59PM. If you will need more time than this to complete your revisions, please reply to this message or contact the journal office at plosone@plos.org. When you're ready to submit your revision, log on to https://www.editorialmanager.com/pone/ and select the 'Submissions Needing Revision' folder to locate your manuscript file.

- A letter that responds to each point raised by the academic editor and reviewer(s). You should upload this letter as a separate file labeled 'Response to Reviewers'.
- A marked-up copy of your manuscript that highlights changes made to the original version. You should upload this as a separate file labeled 'Revised Manuscript with Track Changes'.
- An unmarked version of your revised paper without tracked changes. You should upload this as a separate file labeled 'Manuscript'.

If you would like to make changes to your financial disclosure, please include your updated statement in your cover letter. Guidelines for resubmitting your figure files are available below the reviewer comments at the end of this letter.

If applicable, we recommend that you deposit your laboratory protocols in protocols.io to enhance the reproducibility of your results. Protocols.io assigns your protocol its own identifier (DOI) so that it can be cited independently in the future. For instructions see: https://journals.plos.org/plosone/s/submission-guidelines#loc-laboratory-protocols. Additionally, PLOS ONE offers an option for publishing peer-reviewed Lab Protocol articles, which describe protocols hosted on protocols.io. Read more information on sharing protocols at https://plos.org/protocols?utm_medium=editorial-email&utm_source=authorletters&utm_campaign=protocols.

As the corresponding author, your ORCID iD is verified in the submission system and will appear in the published article. PLOS supports the use of ORCID, and we encourage all coauthors to register for an ORCID iD and use it as well. Please encourage your coauthors to verify their ORCID iD within the submission system before final acceptance, as unverified ORCID iDs will not appear in the published article. *Only*  the individual author can complete the verification step; PLOS staff *cannot*  verify ORCID iDs on behalf of authors.

We look forward to receiving your revised manuscript.

Kind regards,

Emma K. Kalk

Academic Editor

PLOS One

Journal Requirements:

If the reviewer comments include a recommendation to cite specific previously published works, please review and evaluate these publications to determine whether they are relevant and should be cited. There is no requirement to cite these works unless the editor has indicated otherwise.

Please review your reference list to ensure that it is complete and correct. If you have cited papers that have been retracted, please include the rationale for doing so in the manuscript text, or remove these references and replace them with relevant current references. Any changes to the reference list should be mentioned in the rebuttal letter that accompanies your revised manuscript. If you need to cite a retracted article, indicate the article’s retracted status in the References list and also include a citation and full reference for the retraction notice.

[Note: HTML markup is below. Please do not edit.]

Reviewers' comments:

Reviewer's Responses to Questions

**Comments to the Author**

Reviewer #1: All comments have been addressed

Reviewer #2: All comments have been addressed

Reviewer #3: (No Response)

**********

2. Is the manuscript technically sound, and do the data support the conclusions?

Reviewer #1: Yes

Reviewer #2: Yes

Reviewer #3: Yes

**********

3. Has the statistical analysis been performed appropriately and rigorously?

Reviewer #1: Yes

Reviewer #2: Yes

Reviewer #3: Yes

**********

4. Have the authors made all data underlying the findings in their manuscript fully available?

The PLOS Data policy

Reviewer #1: Yes

Reviewer #2: Yes

Reviewer #3: Yes

**********

5. Is the manuscript presented in an intelligible fashion and written in standard English?

Reviewer #1: Yes

Reviewer #2: Yes

Reviewer #3: Yes

**********

Reviewer #1: The authors have sufficiently addressed my feedback, and the paper is now strengthened. It is an important contribution to the literature.

Reviewer #2: I am fully satisfied with the authors responses and the changes made to address my comments. I don't have additional comments.

Reviewer #3: Well and comprehensive written document with technically sound scientific data that supports the conclusions. Findings can be valuable for future public health research focusing on children born to HIV exposed mothers. Just one confirmation - was the equipment use for Bio-impedance analysis (Bodystat 1500 MDD Instrument) calibrated/needed to be calibrated after been transported by motorbike?

**********

what does this mean?). If published, this will include your full peer review and any attached files.

If you choose “no”, your identity will remain anonymous but your review may still be made public.

**Do you want your identity to be public for this peer review?**  For information about this choice, including consent withdrawal, please see our Privacy Policy

Reviewer #1: **Yes:** Elizabeth Widen

Reviewer #2: **Yes:** Paluku Bahwere

Reviewer #3: No

**********

[NOTE: If reviewer comments were submitted as an attachment file, they will be attached to this email and accessible via the submission site. Please log into your account, locate the manuscript record, and check for the action link "View Attachments". If this link does not appear, there are no attachment files.]

To ensure your figures meet our technical requirements, please review our figure guidelines: https://journals.plos.org/plosone/s/figures

You may also use PLOS’s free figure tool, NAAS, to help you prepare publication quality figures: https://journals.plos.org/plosone/s/figures#loc-tools-for-figure-preparation.

NAAS will assess whether your figures meet our technical requirements by comparing each figure against our figure specifications.

# Author comment: Author response to Decision Letter 2

**DOI:** 10.1371/journal.pone.0319542.r004

24 Jul 2026

The term Children Born HIV-free (CBHF) has been updated to children born HIV-exposed uninfected (CHEU) across the manuscript and supporting information.

In addition, one reviewer asked if the bioimpedance machine was calibrated after each visit. For each visit, the Bodystat 1500 was calibrated after being transported by motorbike to the household (using the calibration resistor) and before being tested on the participants. This has been clarified as follows:

“Once set up at the household, calibration was performed before each set of BIA measurements using the 500 Ohm calibration resistor provided (the acceptable range was 497 to 503 Ohms).”

Attachment Submitted filename: 260723_Reviewer comments_PLoS_One.docx

# Editor report: Decision Letter 2

**DOI:** 10.1371/journal.pone.0319542.r005

**Authors:** Emma Kalk

27 Jul 2026

Body composition of children born HIV-exposed uninfected in rural Zimbabwe

PONE-D-25-05396R2

Dear Dr. Piper,

We’re pleased to inform you that your manuscript has been judged scientifically suitable for publication and will be formally accepted for publication once it meets all outstanding technical requirements.

Within one week, you’ll receive an e-mail detailing the required amendments. When these have been addressed, you’ll receive a formal acceptance letter and your manuscript will be scheduled for publication.

An invoice will be generated when your article is formally accepted. Please note, if your institution has a publishing partnership with PLOS and your article meets the relevant criteria, all or part of your publication costs will be covered. Please make sure your user information is up-to-date by logging into Editorial Manager at Editorial Manager® and clicking the ‘Update My Information' link at the top of the page. For questions related to billing, please contact billing support.

If your institution or institutions have a press office, please notify them about your upcoming paper to help maximize its impact. If they’ll be preparing press materials, please inform our press team as soon as possible -- no later than 48 hours after receiving the formal acceptance. Your manuscript will remain under strict press embargo until 2 pm Eastern Time on the date of publication. For more information, please contact onepress@plos.org.

Kind regards,

Emma K. Kalk

Academic Editor

PLOS One

Additional Editor Comments (optional):

Reviewers' comments:

# Editor report: Acceptance letter

**DOI:** 10.1371/journal.pone.0319542.r006

**Authors:** Emma Kalk

PONE-D-25-05396R2

PLOS One

Dear Dr. Piper,

I'm pleased to inform you that your manuscript has been deemed suitable for publication in PLOS One. Congratulations! Your manuscript is now being handed over to our production team.

At this stage, our production department will prepare your paper for publication. This includes ensuring the following:

* All references, tables, and figures are properly cited

* All relevant supporting information is included in the manuscript submission,

* There are no issues that prevent the paper from being properly typeset

You will receive further instructions from the production team, including instructions on how to review your proof when it is ready. Please keep in mind that we are working through a large volume of accepted articles, so please give us a few days to review your paper and let you know the next and final steps.

Lastly, if your institution or institutions have a press office, please let them know about your upcoming paper now to help maximize its impact. If they'll be preparing press materials, please inform our press team within the next 48 hours. Your manuscript will remain under strict press embargo until 2 pm Eastern Time on the date of publication. For more information, please contact onepress@plos.org.

You will receive an invoice from PLOS for your publication fee after your manuscript has reached the completed accept phase. If you receive an email requesting payment before acceptance or for any other service, this may be a phishing scheme. Learn how to identify phishing emails and protect your accounts at https://explore.plos.org/phishing.

If we can help with anything else, please email us at customercare@plos.org.

Thank you for submitting your work to PLOS One and supporting open access.

Kind regards,

PLOS One Editorial Office Staff

on behalf of

Dr. Emma K. Kalk

Academic Editor

PLOS One
